# Lipocalin-2 promotes CKD vascular calcification by aggravating VSMCs ferroptosis through NCOA4/FTH1-mediated ferritinophagy

**DOI:** 10.1038/s41419-024-07260-x

**Published:** 2024-11-29

**Authors:** Yujia Wang, Yuxia Zhang, Min Gao, Zhiqing Chen, Jing Lu, Yongqi Li, Yan Di, Yinan Zhao, Bicheng Liu, Rining Tang

**Affiliations:** Institute of Nephrology, Zhongda Hospital, School of Medicine, Southeast University, Nanjing, China

**Keywords:** Calcification, Genetics research

## Abstract

Vascular calcification (VC) is a common complication of chronic kidney disease (CKD), for which no effective therapies are available. Hyperphosphatemia, a feature of CKD, is a well-known inducer of VC. High phosphate (HP)-induced ferroptosis plays a crucial role in CKD-related VC (CKD-VC), but the mechanisms remain unclear. Lipocalin-2 (LCN2), an iron-trafficking protein, has been implicated in ferroptosis regulation. In the present study, the role of LCN2 as a potential mediator of CKD-VC was investigated. HP-induced LCN2 expression in the arteries of CKD-VC patients, animal models and vascular smooth muscle cells (VSMCs). LCN2 knockout (LCN2KO) mice and wild-type (WT) mice fed with a high adenine and phosphate (AP) diet were studied to explore CKD-VC. Compared with WT mice, LCN2KO mice showed an amelioration of the CKD-VC induced by the AP diet. The inhibition of LCN2 also alleviated HP-induced calcium deposition and phenotypic transition in VSMCs. Conversely, VSMCs-targeted LCN2 overexpression or recombinant LCN2 treatment exacerbated CKD-VC in vivo and in vitro. Mechanistically, nuclear receptor coactivator 4 (NCOA4)/ferritin heavy chain 1 (FTH1)-mediated ferritinophagy-dependent ferroptosis was involved in LCN2-mediated CKD-VC. Under HP conditions, LCN2 interacted with NCOA4, potentially accelerating the degradation of FTH1 and inducing ferroptosis. The inhibition of LCN2 may rescue the degradation of FTH1 and thus ameliorate ferroptosis, significantly suppressing VSMCs calcification. In summary, our study revealed a novel role for LCN2 induced ferritinophagy-dependent ferroptosis in CKD-VC, and targeting LCN2 might be a promising treatment for CKD-VC.

## Introduction

Chronic kidney disease-related vascular calcification (CKD-VC), especially medial calcification, is a significant contributor to cardiovascular morbidity and mortality [1]. Hyperphosphatemia, a common manifestation of mineral metabolic disorders in CKD patients, is a critical factor that drives CKD-VC development [2]. High phosphate (HP) levels promote the osteogenic phenotypic transition of vascular smooth muscle cells (VSMCs), which is a fundamental pathological process in CKD-VC [3]. Nevertheless, the molecular mechanisms underlying the effect of HP on CKD-VC are unclear and to date, there is no effective pharmacological therapy to reverse CKD-VC progression.

Ferroptosis, a newly identified type of programmed cell death induced by iron-dependent accumulation of lipid peroxides, has been proven to play a critical role in hyperlipidaemia-associated VC [4–6]. The findings of a recent study also suggested that ferroptosis could serve as a therapeutic target for the treatment of HP-induced CKD-VC [7]. However, the specific role of ferroptosis in HP-induced CKD-VC, as well as the underlying mechanisms, are largely unknown.

Recent studies have shown that lipocalin-2 (LCN2) is required for ferroptosis [8–10]. LCN2, also known as neutrophil gelatinase-associated lipoprotein (NGAL) or 24p3, is a binding protein responsible for the transportation of lipids and iron. LCN2 also contributes to a range of cardiovascular diseases by activating ferroptosis processes [11–13]. However, the role of LCN2 in calcification-related cardiovascular diseases has been investigated in only a few studies. LCN2 has been reported as a biomarker for atherosclerotic calcification [14]. Pharmacological blockade of LCN2 ameliorates calcific aortic stenosis [15]. Only one clinical study revealed an association between plasma LCN2 level and aortic root calcification in CKD patients, suggesting that LCN2 may play a role in CKD-VC [16]. Despite these aforementioned investigations, the exact pathophysiological role of LCN2 in CKD-VC is still poorly understood.

In this study, we investigated the characteristics and potential mechanisms underlying the role of LCN2 in CKD-VC by performing clinical studies and in vivo and in vitro experiments. Our study is the first to identify the detrimental role of LCN2 in CKD-VC through its activation of HP-induced ferroptosis in VSMCs, thereby providing novel insights into the treatment of CKD-VC.

## Results

### Increased LCN2 levels were associated with the severity of VC in patients with CKD

**Fig. 1:**
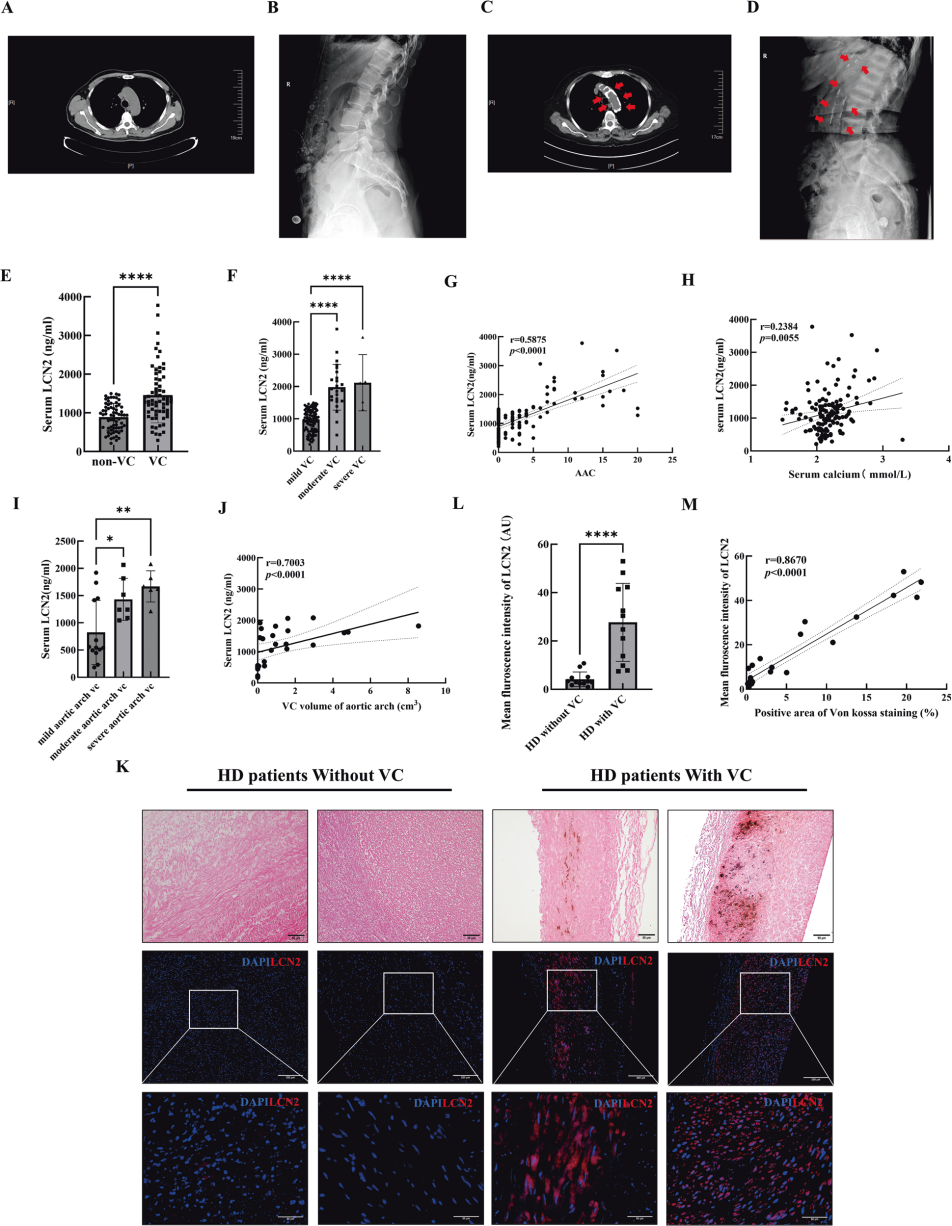
Increased LCN2 levels were associated with the severity of VC in patients with CKD. **A** Representative axial reconstruction of a chest CT scan of a CKD patient without calcification. **B** Representative sagittal abdominal radiography of a CKD patient without calcification. **C** Representative axial reconstruction of a chest CT scan of a CKD patient with aortic arch calcification. **D** Representative sagittal abdominal radiography of a CKD patient with abdominal aorta calcification. The red arrows indicate calcification in the aortic arch and abdominal aorta. **E** Serum LCN2 levels in patients with CKD with VC (n = 66) or without VC (n = 68) according to abdominal radiography. **F** Serum LCN2 levels in subgroups defined by AAC scores (n = 105 for mild VC; n = 24 for moderate VC; n = 5 for severe VC). **G** Scatter diagrams and Spearman coefficient for correlation between serum LCN2 expression level and AAC scores in CKD patients (n = 134). **H** Scatter diagrams and Spearman coefficient for correlation between the serum LCN2 expression level and the serum calcium level in CKD patients (n = 134). **I** Serum LCN2 levels in subgroups defined by aortic arch calcium volume (n = 13 for mild VC; n = 7 for moderate VC; n = 6 for severe VC). **J** Scatter diagrams and Spearman coefficient for correlation between the serum LCN2 expression level and aortic arch calcium volume in CKD patients (n = 26). **K** Upper: Representative Von Kossa staining images of radial arteries from HD patients with or without VC, scale bar: 50 μm. Middle: Immunofluorescence staining images of LCN2 for Vonkossa staining images, scale bar: 200 μm. Bottom: High-magnification images of the immunofluorescence staining images, scale bar: 50 μm. **L** Mean fluorescence intensity of LCN2 in the radial arteries of HD patients with VC (n = 12) or without VC (n = 12). **M** Scatter diagrams and Spearman coefficient for correlation between LCN2 fluorescence intensity and Von Kossa staining-positive areas in the radial arteries of HD patients with or without VC (n = 24). Data are presented as mean ± SD. ^*^p < 0.05, ^**^p < 0.01 and ^****^p < 0.0001. VC vascular calcification, CKD chronic kidney disease, CT computed tomography, AAC abdominal aorta calcification score, HD hemodialyzed.

The chest computed tomography (CT) scans and abdominal radiographs of patients with CKD revealed dense patchy calcifications in the walls of the aortic arch and abdominal aorta (Fig. 1A–D). The baseline clinical and demographic characteristics of the CKD patients and healthy individuals included in this study are presented in Table 1, Table 2 and Supplementary Table 1. CKD patients with abdominal aortic calcification had significantly greater levels of serum LCN2 [1302.01 (1010.30–1856.68) vs. 890.00 ± 353.44, *p* < 0.001; Table 1] than did individuals without VC. The serum LCN2 level progressively increased as VC severity increased (Fig. 1E, F). Serum LCN2 levels were positively correlated with both the abdominal aorta calcification (AAC) score (r = 0.5875, *p* < 0.0001; Fig. 1G) and the serum calcium concentration (r = 0.2384, *p* < 0.01; Fig. 1H).

**Table 1:** Basal characteristics of CKD patients with or without VC according to the AAC score.

	Total (n = 134)	No calcification (n = 68)	Calcification (n = 66)	*P* value
Age, years	51.25 ± 13.92	47.06 ± 12.65	55.58 ± 13.93	**<0.001**
Sex				0.979
Male	77 (57.50%)	39 (57.40%)	38 (57.60%)	
Female	57 (42.50%)	29 (42.60%)	28 (42.40%)	
BMI, kg/m^**2**^	23.33 (20.84-25.63)	23.25 ± 4.23	23.93 ± 4.10	0.368
Serum LCN2, ng/ml	1083.61 (689.65–1396.66)	890.00 ± 353.44	1302.01 (1010.70–1856.68)	**<0.001**
Haemoglobin, g/L	105.57 ± 18.77	101.26 ± 18.25	110.00 ± 18.40	**0.007**
eGFR, mL/min/1.73 m^**2**^	4.72 (3.83–6.36)	4.97 (3.73–6.94)	4.55 (3.85–5.90)	0.295
Urea, mmol/L	21.5 (18.08–26.3)	20.75 (17.23–26.43)	23.16 ± 7.03	0.166
Creatinine, mmol/L	909.46 ± 290.63	885.34 ± 318.52	934.32 ± 258.87	0.331
Ferritin, µg/L	76.50 (33.30–208.03)	85.02 (36.89–184.147)	66.33 (24.61–218.64)	0.731
Iron, µmol/L	13.00 (10.35–16.95)	13.60 (11.00–17.30)	12.20 (9.75–16.70)	0.163
Unsaturated iron, µmol/L	37.14 ± 13.27	36.65 ± 14.25	37.63 ± 12.27	0.673
PTH, pg/mol	208.11 (120.98–418.14)	199.21 (124.12–389.36)	236.97 (84.64–527.30)	0.887
Osteocalcin, ng/ml	175.00 (120.00–221.00)	159.36 ± 70.83	180.50 (128.25–228.00)	0.362
Phosphate, mmol/L	1.86 (1.52–2.39)	1.83 ± 0.48	1.96 (1.54–2.55)	**0.045**
Calcium, mmol/L	2.15 (2.05–2.31)	2.13 (2.04–2.24)	2.23 ± 0.28	**0.023**
Phosphate calcium	3.92 (3.22–5.12)	3.91 ± 1.78	4.29 (3.28–5.66)	**0.018**
Total cholesterol, mmol/L	3.46 ± 0.93	3.41 ± 0.96	3.50 ± 0.91	0.568
Triglycerides, mmol/L	1.35 (0.92–2.17)	1.14 (0.79–1.98)	1.58 (1.15–2.37)	**0.008**
HDL cholesterol, mmol/L	0.87 (0.68–1.13)	0.90 (0.74–1.16)	0.82 (0.60–1.05)	**0.031**
LDL cholesterol, mmol/L	1.53 (1.17–2.05)	1.62 ± 0.63	1.50 (1.21–2.00)	0.801
Lipoprotein A, mg/L	204.50 (99.75–446.00)	201.00 (119.00–418.25)	222.50 (85.25–506.75)	0.712
AAC score	0 (0–4)	0	4 (2.75–8.00)	**<0.001**

The values are expressed as the mean ± SD, median (25–75% quartiles) and n (%) for normally distributed continuous variables, skewed distributed continuous variables and categorical variables, respectively. Statistical significance was assessed via Student’s t test for normally distributed continuous variables or the nonparametric Mann-Whitney U test for skewed distributed continuous variables and chi-square test for categorical variables. Values with *p* < 0.05 were considered statistically significant and are indicated in bold.

*CKD* chronic kidney disease, *VC* vascular calcification, *SD* standard deviation, *BMI* body mass index, *LCN2* lipocalin-2, *eGFR* estimated glomerular filtration rate, *PTH* parathyroid hormone, *HDL-cholesterol* high-density lipoprotein cholesterol, *LDL-cholesterol* low-density lipoprotein cholesterol, *AAC score* abdominal aorta calcification score.

**Table 2:** Basal characteristics of CKD patients with or without VC according to the chest CT.

	Total (n = 26)	No calcification (n = 13)	Calcification (n = 13)	*P* value
Age, years	55.77 ± 17.92	47.23 ± 20.03	64.31 ± 10.48	**0.014**
Sex				0.420
Male	16 (61.5%)	9 (69.2%)	7 (53.8%)	
Female	10 (38.5%)	4 (30.8%)	6 (46.2%)	
BMI, kg/m^**2**^	23.93 (21.64–25.38)	23.94 ± 3.67	24.21 (21.23–25.35)	0.980
Serum LCN2, ng/ml	1181.89 ± 598.85	544.82 (471.42–1431.58)	1540.25 ± 351.88	**0.004**
Haemoglobin, g/L	110.96 ± 21.14	122.16 ± 14.55	99.77 ± 21.15	**0.004**
eGFR, mL/min/1.73 m^**2**^	12.43 (6.00–66.17)	58.16 ± 40.60	6.47 (5.80–12.43)	**0.002**
Urea, mmol/L	13.65 (7.85–23.20)	8.00 (5.75–13.35)	20.85 ± 8.00	**0.001**
Creatinine, mmol/L	385.50 (111.50–716.75)	112.00 (71.00–441.00)	630.62 ± 267.74	**0.004**
Ferritin, µg/L	83.48 (47.72–229.10)	99.06 (28.81–719.88)	79.67 (47.67–189.11)	0.805
Iron, µmol/L	11.32 ± 5.22	13.72 ± 6.94	10.21 ± 4.08	0.290
Unsaturated iron, µmol/L	32.40 (28.60–40.30)	28.85 (25.68–44.48)	37.57 ± 10.07	0.219
PTH, pg/mol	169.71 (32.38–336.24)	32.81 (30.00–137.00)	282.71 (148.73–368.26)	**0.010**
Calcium, mmol/L	2.14 (1.93–2.29)	2.15 ± 0.22	2.07 (1.86–2.28)	0.343
Phosphate, mmol/L	1.62 ± 0.60	1.31 ± 0.25	1.92 ± 0.70	**0.009**
Phosphate calcium	3.04 (2.47–3.99)	2.80 ± 0.56	4.00 ± 1.34	**0.006**
Total cholesterol, mmol/L	3.78 (2.45–5.24)	4.91 ± 2.25	3.43 ± 1.36	0.053
Triglycerides, mmol/L	1.49 (1.02–2.09)	1.44 ± 0.71	1.49 (1.37–2.04)	0.427
HDL cholesterol, mmol/L	1.04 (0.63–1.15)	1.25 ± 0.65	0.79 ± 0.25	**0.025**
LDL cholesterol, mmol/L	1.88 (1.08–3.57)	2.97 ± 1.76	1.65 ± 0.92	**0.025**
Lipoprotein A, mg/L	155.00 (66.50–493.50)	105.00 (64.50–434.00)	338.62 ± 289.13	0.590
Aortic arch calcium volume, cm^**3**^	0.54 (0.05–1.60)	0.06 (0.00–0.23)	1.60 (0.95–3.78)	**<0.001**

The values are expressed as mean ± SD, median (25–75% quartiles) and n (%) for normally distributed continuous variables, skewed distributed continuous variables and categorical variables, respectively. Statistical significance was assessed via Student’s t test for normally distributed continuous variables or the nonparametric Mann-Whitney U test for skewed distributed continuous variables and chi-square test for categorical variables. Values with *p* < 0.05 were considered statistically significant and are indicated in bold. The calcification volume was quantified by extracting the aortic arch calcium with density ≥130 HU from the volume-rendered image with the assistance of semiautomatic software.

*CKD* chronic kidney disease, *VC* vascular calcification, *SD* standard deviation, *BMI* body mass index, *LCN2* lipocalin-2, *eGFR* estimated glomerular filtration rate, *PTH* parathyroid hormone, *HDL-cholesterol* high-density lipoprotein cholesterol, *LDL-cholesterol* low-density lipoprotein cholesterol.

Given the significant correlation between LCN2 levels and the estimated glomerular filtration rate (eGFR) [17], we analysed the correlation between serum LCN2 levels and CKD-VC in CKD stage 1-3 non-VC patients and end-stage renal damage (ESRD) VC patients. Serum LCN2 [1540.25 ± 351.88 vs. 544.82 (471.42–1431.58), *p* < 0.01; Table 2] was significantly increased in ESRD patients with VC. The serum LCN2 level increased as aortic arch calcification progressed (Fig. 1I). A positive correlation was also observed between the serum LCN2 level and the aortic arch calcium volume in these patients (r = 0.7003, *p* < 0.0001; Fig. 1J).

To confirm the correlation between LCN2 expression and CKD-VC, immunofluorescence (IF) staining for LCN2 was performed in the radial arteries of hemodialyzed (HD) patients. The baseline clinical and demographic characteristics of the HD patients included in this study are presented in Table 3. As shown in Fig. 1K, L, LCN2 expression in the tunica media of the radial arteries was markedly higher in HD patients with VC than in individuals without VC. Notably, the increase in LCN2 level was positively correlated with the area of calcification quantified by Von Kossa staining (r = 0.8670, *p* < 0.0001; Fig. 1M). Taken together, these data indicate that LCN2 expression was increased in VC patients and was correlated with VC severity among patients with CKD.

**Table 3:** Baseline characteristics of HD patients with or without VC according to Von Kossa staining of the radial arteries.

	Total (n = 24)	No calcification (n = 12)	Calcification (n = 12)	*P* value
Age, years	55.32 ± 13.76	58.08 ± 13.47	53.75 ± 14.28	0.453
Sex				**0.041**
Male	13 (54.2%)	9 (75%)	4 (33.3%)	
Female	11 (45.8%)	3 (25%)	8 (66.7%)	
BMI, kg/m^**2**^	22.93 ± 4.63	22.17 ± 3.63	22.59 ± 5.55	0.841
LCN2,AU	8.61 (3.05–29.00)	3.14 (2.42–5.03)	27.41 ± 16.13	**<0.001**
Haemoglobin, g/L	101.13 ± 18.93	111.50 (88.75–120.00)	98.25 ± 17.01	0.326
eGFR, mL/min/1.73 m^**2**^	4.88 (4.29–6.33)	5.34 ± 1.70	4.88 (4.30–6.31)	1.000
Urea, mmol/L	21.85 ± 8.70	23.53 ± 10.70	20.17 ± 6.13	0.357
Creatinine, mmol/L	829.04 ± 242.74	878.75 ± 277.86	779.33 ± 201.52	0.327
Ferritin, µg/L	99.87 (39.11–251.12)	109.39 (33.41–251.12)	83.94 (39.11–223.56)	0.817
Iron, µmol/L	9.70 (8.20–16.15)	9.70 (8.83–16.15)	11.86 ± 6.96	0.665
Unsaturated iron, µmol/L	36.20 ± 14.20	34.78 ± 12.51	37.62 ± 16.14	0.636
PTH, pg/mol	196.71 (140.19–303.51)	200.39 (182.07–376.06)	177.61 ± 115.86	0.204
Calcium, mmol/L	2.28 ± 0.24	2.26 ± 0.25	2.30 ± 0.24	0.685
Phosphate, mmol/L	1.76 ± 0.74	1.92 ± 0.93	1.59 ± 0.46	0.278
Phosphate calcium	3.65 (2.71–5.05)	4.44 ± 2.40	3.64 ± 1.05	0.306
Total cholesterol, mmol/L	3.40 ± 1.18	3.14 ± 1.03	3.67 ± 1.29	0.280
Triglycerides, mmol/L	1.53 (1.15–2.13)	1.44 (1.00–2.13)	1.54 (1.36–2.39)	0.436
HDL cholesterol, mmol/L	0.93 ± 0.39	0.95 (0.63–1.40)	0.78 (0.63–1.12)	0.488
LDL cholesterol, mmol/L	1.67 ± 0.70	1.72 (0.84–1.95)	1.87 ± 0.75	0.312
Lipoprotein A, mg/L	292.50 (161.50–495.25)	308.00 (182.75–495.25)	203.50 (84.25–478.25)	0.312
Von Kossa staining postive area (%)	1.23 (0.49–9.92)	0.47 ± 0.19	11.06 ± 7.62	**<0.001**

The values are expressed as mean ± SD, median (25% to 75% quartiles) and n (%) for normally distributed continuous variables, skewed distributed continuous variables and categorical variables, respectively. Statistical significance was assessed via Student’s t test for normally distributed continuous variables or the nonparametric Mann-Whitney U test for skewed distributed continuous variables and chi-square test for categorical variables. Values with *p* < 0.05 were considered statistically significant and are indicated in bold.

*HD* hemodialyzed, *VC* vascular calcification, *SD* standard deviation, *BMI* body mass index, *LCN2* lipocalin-2, *AU* augmentation unit, *eGFR* estimated glomerular filtration rate, *PTH* parathyroid hormone, *HDL-cholesterol* high-density lipoprotein cholesterol, *LDL-cholesterol* low-density lipoprotein cholesterol.

### The LCN2 expression level was elevated under CKD-VC conditions both in vivo and in vitro

**Fig. 2:**
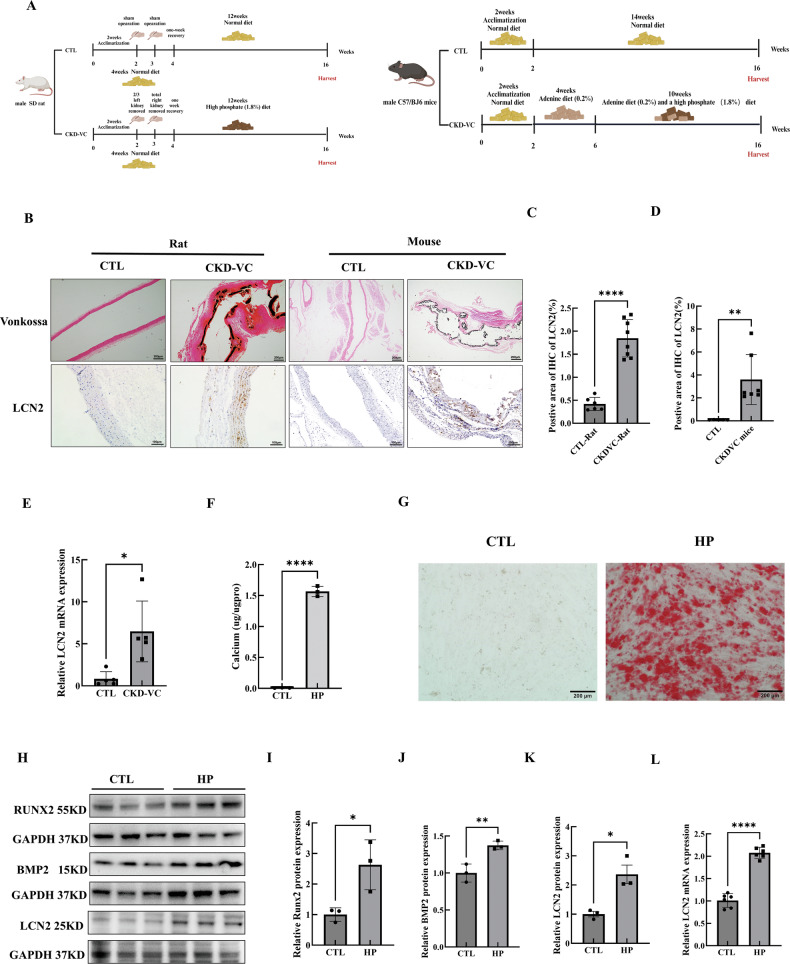
The LCN2 expression level was increased in two CKD-VC model animals and in HP-stimulated VSMCs. **A** Scheme of the 5/6 nephrectomy and HP diet-induced CKD-VC rat model and AP diet-induced CKD-VC mouse model. **B** Representative Von Kossa staining and IHC images of LCN2 in the aortic smooth muscle layer from CKD-VC model rats and mice. Scale bar: 200 μm for Von Kossa staining, 100 μm for IHC images. **C** Quantification of the LCN2-positive area in CKD-VC model rats (n = 6 for the CTL group; n = 8 for the CKD-VC model group). **D** Quantification of the LCN2-positive area in CKD-VC model mice (n = 5 for the CTL group; n = 7 for the CKD-VC model group). **E** The expression of LCN2 mRNA in the aortas of CTL and CKD-VC rats was analysed by qRT-PCR (n = 5 per group). Mouse VSMCs were treated with growth medium (CTL) or calcifying medium containing 3.0 mM HP sodium for 5 days. Calcium deposition in VSMCs was assessed by a calcium concentration assay normalised to the protein concentration (n = 3 per group) (**F**) and alizarin red staining (**G**) (positive staining: red; scale bar: 200 μm). **H**–**K** The protein levels of osteogenic phenotype markers (RUNX2, BMP2) and LCN2 were determined via Western blot (n = 3 per group). **L** LCN2 mRNA expression in VSMCs exposed to HP (3.0 mM) (n = 6 per group). Data are presented as mean ± SD. ^*^p < 0.05, ^**^p < 0.01, ^***^p < 0.001 and ^****^p < 0.0001. CKD-VC chronic kidney disease-related vascular calcification, HP high phosphate, VSMCs vascular smooth muscle cells, IHC immunohistochemical, AP adenine and phosphate, CTL control, qRT-PCR quantitative real-time polymerase chain reaction, RUNX2 runt-related transcription factor 2, BMP2 bone morphogenetic protein 2, SD standard deviation.

To further verify the observed LCN2 levels in vivo, two CKD-VC animal models were evaluated (Fig. 2A, Supplementary Fig. 1A–K). Immunohistochemical (IHC) results showed that LCN2 expression levels in the aortic smooth muscle layers were greater in CKD-VC rats and mice than that in control group rats and mice (Fig. 2B–D). Increased LCN2 mRNA level was also observed in the arteries of CKD-VC rats (Fig. 2E). To investigate LCN2 expression in CKD-VC in vitro, VSMCs were cultured in calcifying medium supplemented with 3 mM inorganic phosphate (high phosphate, HP) for 5 days. An intracellular calcium assay and alizarin red staining revealed calcium deposition in the VSMCs (Fig. 2F, G). Notably, Western blot analysis and quantitative real-time polymerase chain reaction (qRT‒PCR) revealed the osteogenic phenotypic transition of VSMCs (Fig. 2H–J, Supplementary Fig. 1L–O). Consistent with the increase in osteogenic marker expression, the protein and mRNA expression levels of LCN2 were also significantly increased in VSMCs following HP stimulation (Fig. 2H, K, L). Taken together, these results indicate that the expression of LCN2 was increased under CKD-VC conditions both in vivo and in vitro.

### LCN2 deletion attenuated CKD-VC in vivo

**Fig. 3:**
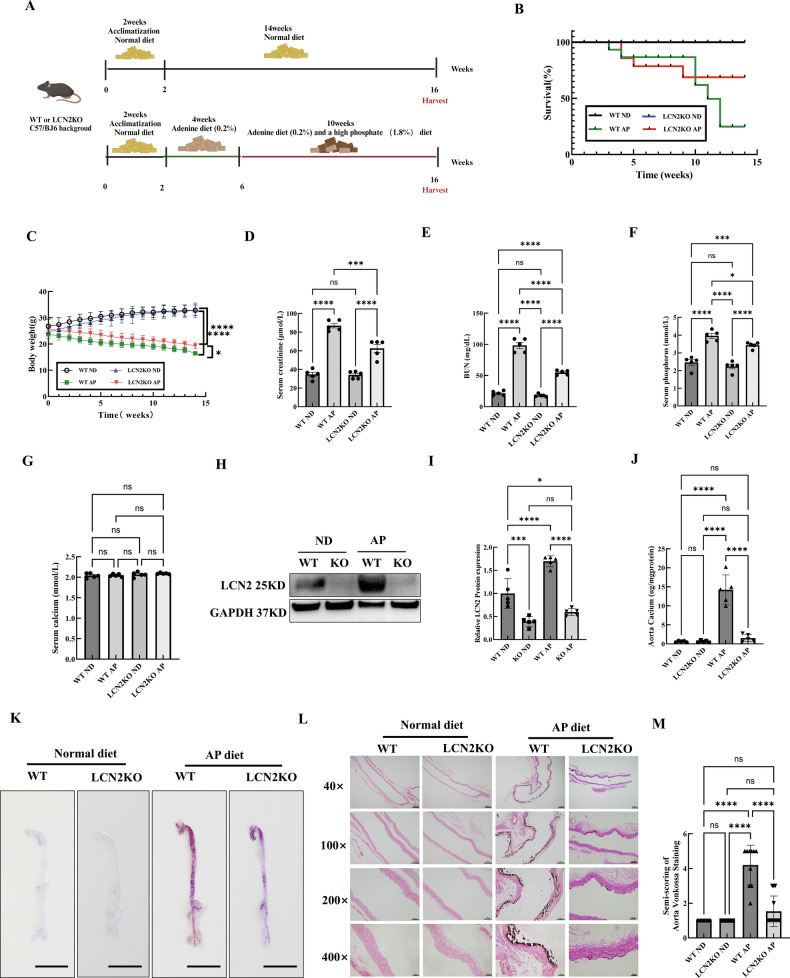
Deletion of LCN2 attenuated AP diet-induced CKD-VC in mice. **A** Scheme of the experiment. After 2 weeks of acclimatisation, LCN2KO and WT mice were fed with a high adenine and phosphate diet (AP) or a normal diet (ND) for 14 weeks. **B** Survival curves of the experimental mice. Body weights (**C**), serum creatinine (**D**), BUN (**E**), serum phosphorus (**F**) and serum calcium levels (**G**) of the four groups (n = 5 per group). **H** Western-blot analysis and quantification (**I**) of LCN2 in the aorta (n = 5 per group). **J** Calcium concentrations of aortas (n = 5 per group). **K** Representative alizarin red stained images of the aorta. Scale bar:1 cm. **L** Representative Von Kossa staining of the aortic smooth muscle layer in the four groups and semi-quantification (**M**). Scale bar: 200 μm for 40×; 100 μm for 100×; 50 μm for 200×, 20 μm for 400×. Data are presented as mean ± SD. ^*^p < 0.05, ^**^p < 0.01, ^***^p < 0.001 and ^****^p < 0.0001. CKD-VC chronic kidney disease-related vascular calcification, AP adenine and phosphate, KO knockout, WT wild type, BUN blood urea nitrogen, SD standard deviation.

LCN2 knockout (LCN2KO) and wild-type (WT) mice were fed a high adenine and phosphate diet (AP) to induce CKD-VC (Fig. 3A). The LCN2KO and WT mice in the control group were fed a normal diet (ND). LCN2 deletion increased survival and ameliorated body weight loss in AP diet-induced CKD-VC mice (Fig. 3B, C). The serum renal function indices significantly increased after AP diet induction of CKD-VC but were notably lower in the LCN2KO AP group than in the WT AP group (Fig. 3D, E). Furthermore, the serum level of phosphorus was significantly lower in LCN2KO mice than in WT mice after AP diet induction of CKD-VC, whereas the serum calcium level was unchanged (Fig. 3F, G). LCN2 deletion in the aorta was validated by Western blot analysis (Fig. 3H, I). LCN2 deletion significantly attenuated aortic calcification, as indicated by the calcium concentration analysis, alizarin red staining and Von Kossa staining (Fig. 3J–M).

### LCN2 knockdown or inhibition in vitro alleviated HP-induced VSMCs calcification

**Fig. 4:**
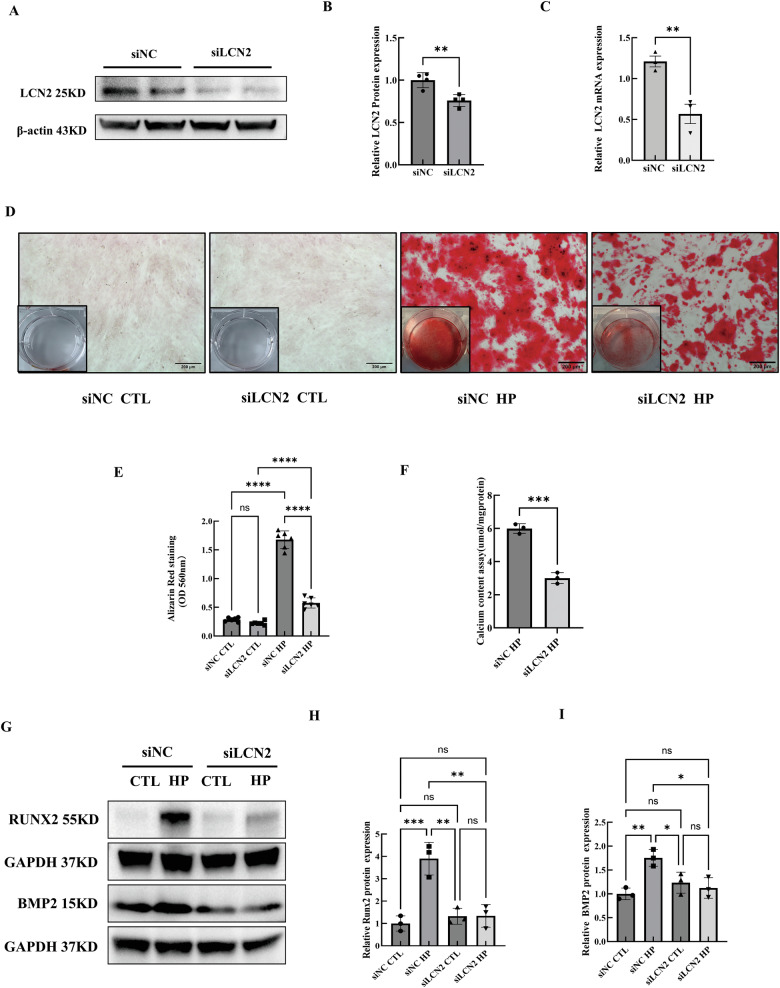
Knockdown of LCN2 alleviates HP-induced VSMCs calcification. **A**–**C** VSMCs were transfected with LCN2 siRNA (siLCN2) or NC siRNA (siNC). The efficacy of transfection was verified by Western blot (n = 4 per group) and qRT-PCR (n = 3 per group). **D** Calcium deposition in VSMCs was assessed by Alizarin red staining (positive staining: red; scale bar: 200 μm) and quantified using hexadecylpyridinium chloride at an OD of 560 nm (**E**) (n = 6 per group). **F** Calcium deposition in VSMCs was assessed by calcium concentration assay and was normalised to the protein concentration (n = 3 per group). **G** Western blot analysis and quantification of the RUNX2 (**H**) and BMP2 (**I**) protein expression in siNC or siLCN2-transfected VSMCs exposed to HP (3.0 mM) or growth medium (CTL) (n = 3 per group). Data are presented as mean ± SD. ^*^p < 0.05, ^**^p < 0.01, ^***^p < 0.001 and ^****^p < 0.0001. qRT-PCR quantitative real-time polymerase chain reaction, HP high phosphate, VSMC vascular smooth muscle cell, siRNA small interfering RNA, NC negative control, OD optical density, CTL control, RUNX2 runt-related transcription factor 2, BMP2 bone morphogenetic protein 2, SD standard deviation.

VSMCs were transfected with LCN2 small interfering RNA (siLCN2) to knockdown LCN2 or negative control small interfering RNA (siNC) (Fig. 4A–C). Alizarin red staining and calcium concentration assay revealed that LCN2 knockdown markedly alleviated VSMCs calcification in the presence of HP on day 5 (Fig. 4D–F). Additionally, LCN2 knockdown inhibited the osteogenic phenotypic transition of VSMCs, as indicated by changes in the expression of osteogenic markers (Fig. 4G–I).

During in vitro culture of VSMCs, the addition of 0.5 µmol/L of the LCN2 inhibitor ZINC00784494 led to a significant downregulation of LCN2 expression in VSMCs (Supplementary Fig. 2A, B). Additionally, Alizarin red staining and quantification revealed that 0.5 µmol/L ZINC00784494 significantly mitigated the degree of HP-induced VSMCs calcification (Supplementary Fig. 2C, D).

### LCN2 overexpression in vivo and in vitro exacerbated CKD-VC

**Fig. 5:**
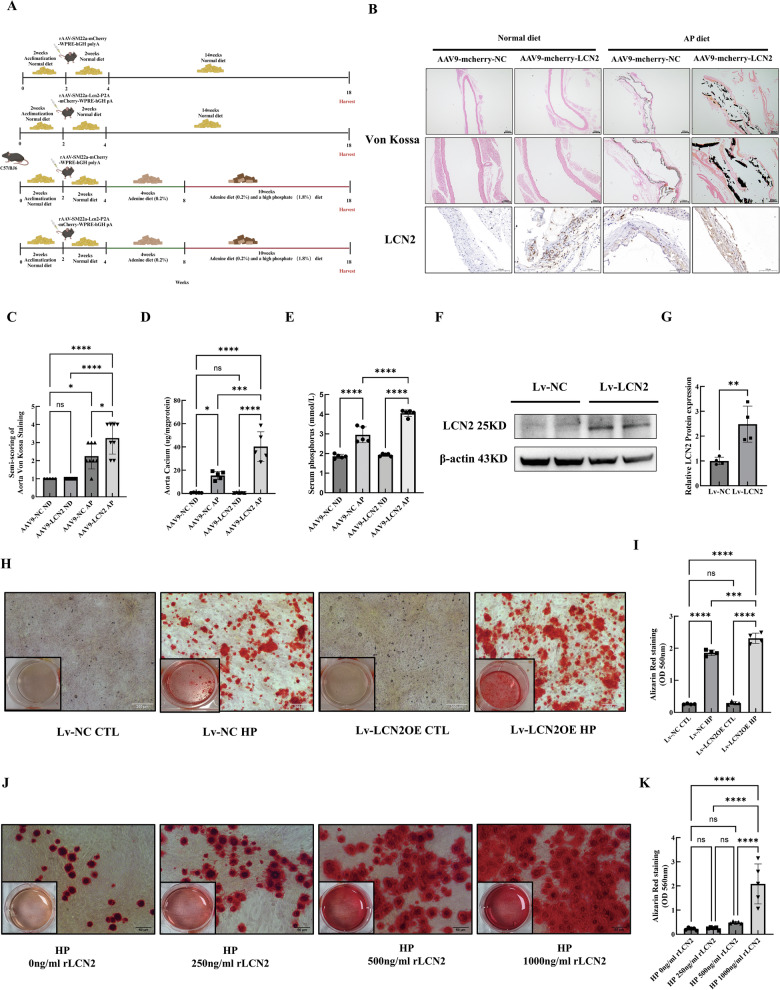
LCN2 overexpression exacerbated CKD-VC in vivo and in vitro. **A** Scheme of the experiment. Mice were injected with AAV9-SM22α-vector and AAV9-SM22α- LCN2 and then provided with an AP diet or normal diet (ND) for 14 weeks. **B** Upper and middle: representative images of Von Kossa staining of the aortic smooth muscle layer. Scale bar: 200 μm for upper; 100 μm for middle; bottom: IHC images of LCN2 expression in four groups. Scale bar: 100 μm. **C** Semi-quantification of positive Von Kossa staining of the aortic smooth muscle layer **D** Calcium concentrations of the aorta (n = 5 per group). **E** Serum phosphorus levels (n = 5 per group). **F**, **G** LCN2 overexpression efficiency was validated by Western blot analysis (n = 4 per group). **H** Calcium deposition in LCN2-overexpressing VSMCs in growth medium or HP conditions was assessed by alizarin red staining (positive staining: red; scale bar: 200 μm) and quantified by hexadecylpyridinium chloride (n = 4 per group) (**I**). **J** Representative images of alizarin red staining of VSMCs treated with different doses of rLCN2 (0, 250, 500, or 1000 ng/ml) in the presence of HP. Scale bar: 50 μm. **K** Quantification of the alizarin red staining by hexadecylpyridinium chloride (n = 5 per group). Data are presented as mean ± SD. ^*^p < 0.05, ^**^p < 0.01, ^***^p < 0.001 and ^****^p < 0.0001. AAV adeno-associated virus, Lv lentivirus, CKD-VC chronic kidney disease-related vascular calcification, AP adenine and phosphate, ND normal diet, IHC immunohistochemical, VSMCs vascular smooth muscle cells, HP high phosphate, rLCN2 recombinant LCN2, SD standard deviation.

To evaluate whether LCN2 overexpression exacerbates CKD-VC progression in vivo, we established a VSMCs-targeted LCN2 overexpressing mouse model by injecting adeno-associated virus AAV9-SM22α-vector or AAV9-SM22α-LCN2 into mice through the lateral tail vein and then providing the mice an AP diet to induce CKD-VC (Fig. 5A). As expected, VSMCs-targeted LCN2 overexpression exacerbated CKD-VC, as determined by Von Kossa staining, calcium concentration assay and serum phosphorus level analysis (Fig. 5B–E). Next, we explored the effect of lentivirus-mediated LCN2 overexpression on VSMCs calcification in vitro. LCN2 overexpression efficiency is shown in Fig. 5F, G. LCN2 overexpression exacerbated HP-induced VSMCs calcification, as determined by alizarin red staining and quantification (Fig. 5H, I). Next, we treated VSMCs with different concentrations of recombinant LCN2 (rLCN2) under HP conditions. HP-induced calcification of VSMCs progressively worsened with increasing concentrations of rLCN2 and 1000 ng/ml rLCN2 significantly exacerbated VSMCs calcification, as determined by alizarin red staining and quantitative analysis (Fig. 5J, K). Collectively, these results demonstrate that LCN2 exacerbated CKD-VC in vivo and in vitro.

### Ferritinophagy-dependent ferroptosis was a critical contributor to CKD-VC

**Fig. 6:**
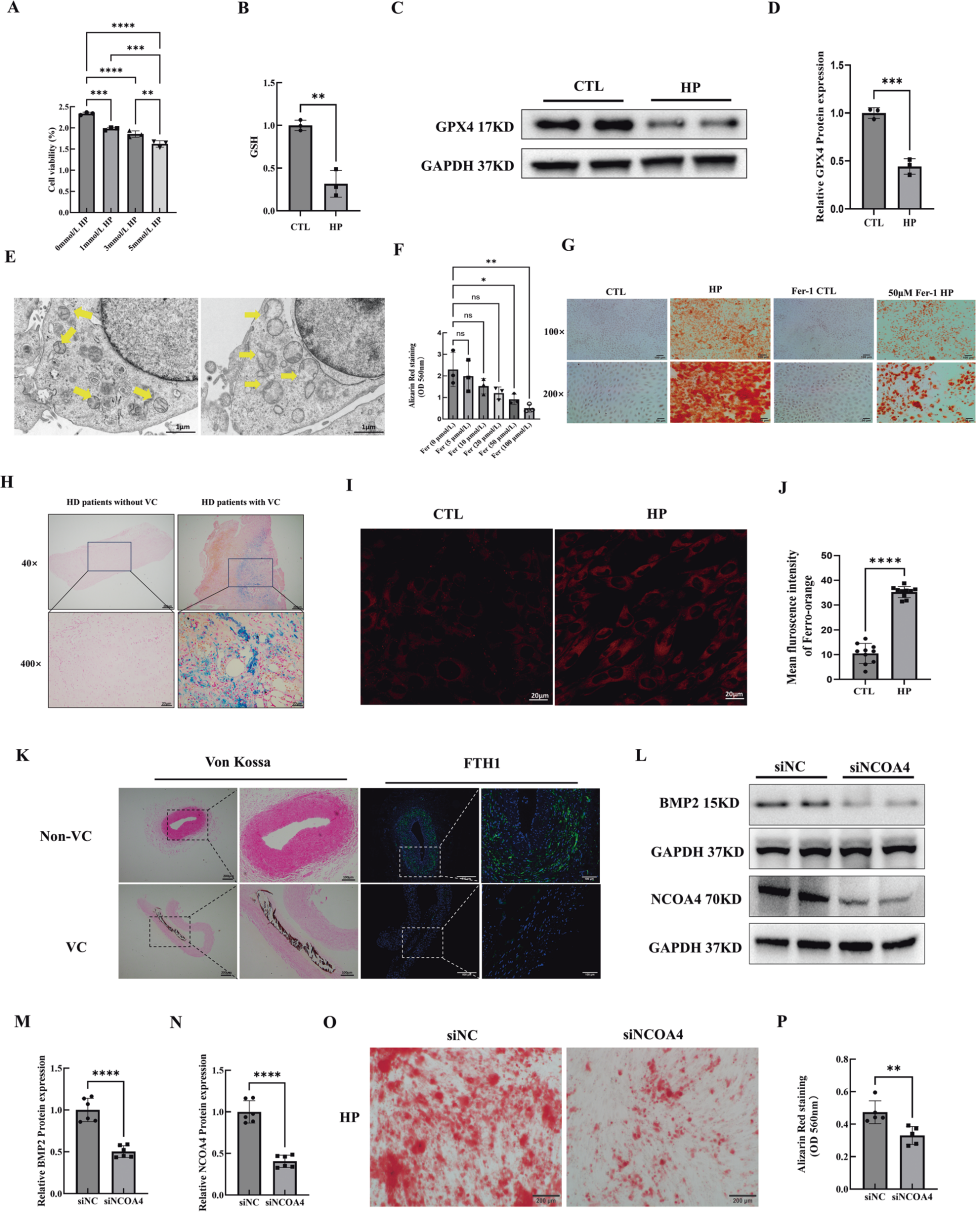
Ferritinophagy-dependent ferroptosis served as a critical contributor to CKD-VC. **A** Cell viability of VSMCs after treatment with different concentrations of (0, 1, 3, 5 mmol/L) phosphate sodium, as determined via the CCK-8 assay. **B** GSH content in VSMCs after HP treatment or growing medium (CTL) treatment. **C** Representative western-blot bands and quantification (**D**) of GPX4 expression in HP-treated VSMCs. **E** Typical features of ferroptosis in the mitochondrial morphology of VSMC subjected to HP treatment. (yellow arrows: mitochondria; scale bar: 1 µm). **F** Quantification of alizarin red staining of VSMCs treated by different doses of Fer-1 (5,10, 20, 50, or 100 µmol/L) under HP conditions. **G** Representative images of alizarin red staining of 50 µmol/L Fer-1-treated VSMCs in the absence or presence of HP. Scale bar:100 μm for 100×; 50 μm for 200×. **H** Prussian blue iron staining of radial artery tissue sections from HD patients (positive staining: blue; scale bar 200 µm for 40× and 20 µm for 400×). **I** Representative fluorescence images of the iron probe FerroOrange in VSMCs in the absence or presence of HP. Scale bar:20 µm. **J** Quantification of fluorescence intensity of FerroOrange. (n = 10 per group). **K** Representative Von Kossa staining images and immunofluorescence image of FTH1 in the calcifying radial arteries of HD patients. Scale bar: 200 μm for low-magnification Von Kossa staining images; 200 μm for high-magnification Von Kossa staining images; 500 μm for low-magnification immunofluorescence images; 100 μm for high-magnification immunofluorescence images. **L** Representative Western blot images and quantification of BMP2 (**M**) and NCOA4 (**N**) expression in NCOA4-knockdown VSMCs under HP conditions (n = 6 per group). Representative images (**O**) and quantification (**P**) of alizarin red staining in NCOA4-knockdown VSMCs under HP conditions. Data are presented as mean ± SD. ^*^p < 0.05, ^**^p < 0.01, ^***^p < 0.001 and ^****^p < 0.0001. CKD-VC chronic kidney disease-related vascular calcification, VSMCs vascular smooth muscle cells, HP high phosphate, CCK-8 Cell Counting Kit-8, CTL control, GSH glutathione, GPX4 glutathione peroxidase 4, Fer-1 ferrostain-1, HD hemodialyzed.

A previous study revealed that the mechanism underlying the role of HP conditions in CKD-VC involves ferroptosis [7]. Therefore, we explored the viability and lipid oxidation levels of HP-stimulated VSMCs. Cell Counting Kit-8 (CCK-8) assay revealed that HP conditions triggered VSMCs toxicity in a dose-dependent manner (Fig. 6A). The glutathione (GSH) content and glutathione peroxidase 4 expression levels were significantly decreased in HP-treated VSMCs (Fig. 6B–D). To further determine the occurrence of ferroptosis, we examined the mitochondrial structure in VSMCs via transmission electron microscopy (TEM). Notably, HP conditions significantly altered the mitochondrial structure, with a reduction in the number of mitochondrial cristae and the rupture of the mitochondrial membrane (Fig. 6E). Next, we treated VSMCs with different concentrations of a specific ferroptosis inhibitor, ferrostatin-1 (Fer-1). Gradual attenuation of VSMCs calcification with increasing doses of Fer-1 was observed. Furthermore, 50 µM or 100 µM Fer-1 significantly attenuated VSMCs calcification (Fig. 6F, G). Taken together, these data confirm that ferroptosis served as a critical contributor to the development of HP-induced CKD-VC.

To further determine the iron concentration in the radial arteries of CKD patients, we performed Prussian blue iron staining of radial artery samples obtained from HD patients. Iron-positive staining areas were abundant in the arteries of HD patients with severe VC (Fig. 6H). Next, we explored the cellular labile iron (Fe^2+^) pool in HP-treated VSMCs with the fluorescent probe FerroOrange. The fluorescence signal of FerroOrange increased in the VSMCs after HP treatment, indicating an increase in the level of intracellular free iron in the HP-treated VSMCs. (Fig. 6I, J). Intracellular iron accumulation has been reported to result from abnormal autophagic degradation of ferritin, a process also known as ferritinophagy [18]. To date, the relationship between ferritinophagy and HP-induced CKD-VC has not been explored. Thus, we examined ferritin heavy chain (FTH1) expression in the radial arteries of HD patients. FTH1 expression was notably decreased in HD patients with VC (Fig. 6K). To clarify the relationship between ferritinophagy and HP-induced CKD-VC, VSMCs were transfected with siRNA to knock down nuclear receptor coactivator 4 (NCOA4), a key regulator of ferritinophagy. NCOA4 knockdown mitigated osteogenic remodelling in VSMCs under HP conditions (Fig. 6L–N) and significantly attenuated HP-induced calcium deposition in VSMCs (Fig. 6O, P). Taken together, our results indicate that ferritinophagy-dependent ferroptosis was a critical contributor to CKD-VC.

### LCN2 contributed to CKD-VC by modulating ferritinophagy-dependent ferroptosis through NCOA4/FTH1 signalling

**Fig. 7:**
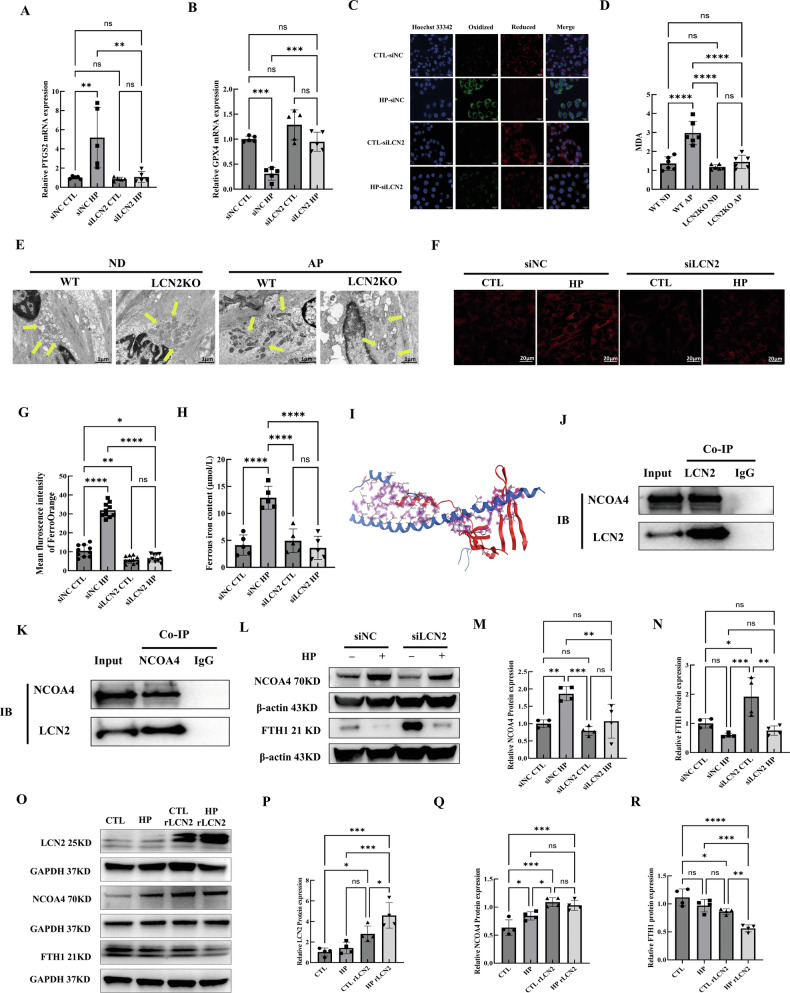
Knockdown of LCN2 ameliorated CKD-VC by modulating NCOA4/FTH1-mediated ferritinophagy-dependent ferroptosis. **A** Representative mRNA expression of PTGS2 in LCN2-knockdown VSMCs under HP or growth medium conditions (n = 5 per group). **B** Representative mRNA expression of GPX4 in LCN2-knockdown VSMCs under HP or growth medium conditions (n = 5 per group). **C** Lipid ROS levels were evaluated by C11-BODIPY^581/591^ staining. Scale bar: 20 µm. (**D**) MDA content in the aorta. (n = 6 per group). **E** Typical features of ferroptosis-related changes in the mitochondrial morphology of aortic tissues from mice. (yellow arrows: mitochondria; scale bar:1 µm). **F** Representative fluorescence images and mean fluorescence intensity (**G**) of the iron probe FerroOrange in LCN2-knockdown VSMCs in the absence or presence of HP (n = 10 per group). Scale bar: 20 µm. **H** Ferrous iron concentrations in LCN2-knockdown VSMCs in the absence or presence of HP (n = 5 per group). **I** Molecular docking of the LCN2 protein and NCOA4 protein was performed with clu-spro2.0. The blue colour represents the structure of LCN2, and the red colour represents the molecular structure of NCOA4. **J**, **K** VSMCs lysates were immunoprecipitated with anti-LCN2 or anti-NCOA4 antibodies and then analysed by immunoblotting with anti-LCN2 and anti-NCOA4 antibodies. **L** Representative Western blot images and quantification of NCOA4 (**M**) and FTH1 (**N**) protein expression levels in LCN2- knockdown VSMCs in the absence or presence of HP (n = 4 per group). **O** Representative Western blot images and quantification of LCN2 (**P**), NCOA4 (**Q**) and FTH1 protein expression (**R**) in the recombinant LCN2-treated VSMCs in the presence or absence of HP (n = 4 per group). Data are presented as mean ± SD. ^*^p < 0.05, ^**^p < 0.01, ^***^p < 0.001 and ^****^p < 0.0001. CKD-VC chronic kidney disease-related vascular calcification, VSMC vascular smooth muscle cell, HP high phosphate, GPX4 glutathione peroxidase 4, ROS reactive oxygen species, MDA malondialdehyde, SD standard deviation.

LCN2 has been implicated in ferroptosis [8–10]. Therefore, we explored the role of LCN2 in regulating HP-induced VSMCs ferroptosis. LCN2 knockdown attenuated HP-induced VSMCs ferroptosis through the regulation of pro-ferroptotic PTGS2 mRNA and anti-ferroptotic GPX4 mRNA expression (Fig. 7A, B). LCN2 knockdown reduced intracellular lipid reactive oxygen species (ROS) in HP-stimulated VSMCs (Fig. 7C). LCN2 depletion significantly decreased the content of malondialdehyde (MDA), a lipid peroxidation byproduct, in the calcified aortas of AP diet-fed CKD-VC mice (Fig. 7D). Additionally, LCN2 depletion ameliorated ferroptosis-associated changes in mitochondrial morphology in mouse aortic tissue (Fig. 7E). These data suggest that LCN2 played a role in CKD-VC by modulating VSMCs ferroptosis.

Next, we investigated the relationship between LCN2 and HP-induced ferritinophagy. The knockdown of LCN2 reduced the labile iron (Fe^2+^) pool in HP-stimulated VSMCs, as indicated by the fluorescent probe FerroOrange staining images (Fig. 7F, G). We also found that lentivirus-mediated LCN2 overexpression or recombinant LCN2 treatment increased the ferrous iron (Fe^2+^) pool in HP-stimulated VSMCs (Supplementary Fig. 3C, F). Consistently, the iron concentration assay also revealed that LCN2 knockdown notably reduced the free iron (Fe^2+^) concentration in HP-stimulated VSMCs (Fig. 7H), suggesting that LCN2 regulates NCOA4-mediated ferritinophagy in VSMCs exposed to HP conditions. The interaction between the LCN2 and NCOA4 proteins was explored through molecular docking. From the numerous outcomes generated by ClusPro 2.0, the complex exhibiting the strongest binding affinity was chosen for visualisation (Fig. 7I). Next, we performed co-immunoprecipitation (co-IP) and Western blot experiments to verify the above findings. The protein containing the LCN2 primary antibody bound to NCOA4, whereas the protein containing IgG had minimal binding (Fig. 7J). We also detected the LCN2 protein in the sample containing the primary antibody against NCOA4, but the LCN2 protein concentration in the IgG sample was very low (Fig. 7K). These findings suggested that LCN2 might bind to NCOA4 in a tight manner, which was in line with the results of the molecular docking analysis. Notably, the knockdown of LCN2 reversed the HP-induced increase in NCOA4 expression in VSMCs and the subsequent decrease in FTH1 expression in VSMCs (Fig. 7L, N). Consistently, recombinant LCN2 treatment further exacerbated the upregulated expression of NCOA4 in HP-stimulated VSMCs (Fig. 7O–R).

## Discussion

CKD-VC has drawn great attention recently because of its high mortality and morbidity. According to multicenter prospective cohort studies [19, 20], HP is a major contributor to the progression of CKD-VC and the poor outcomes of affected patients. Through this study, we provide the first evidence that HP conditions triggered an increase in LCN2 expression in the VSMCs and vascular walls of CKD patients and animal models. Furthermore, excessive LCN2 levels contributed to the development of CKD-VC. Mechanistically, the inhibition of LCN2 effectively mitigated CKD-VC by regulating ferritinophagy-dependent ferroptosis in VSMCs. Our findings demonstrated that HP conditions triggers NCOA4/FTH1 axis-mediated ferritinophagy-dependent ferroptosis in VSMCs. The inhibition of LCN2 significantly decreased NCOA4 protein levels and restored FTH1 protein levels to protect VSMCs from HP-induced ferroptosis and calcification. Therefore, strategies targeting LCN2 may hold therapeutic promise in the treatment of CKD-VC.

LCN2, also known as neutrophil gelatinase-associated lipocalin, has been extensively studied as a reliable biomarker for acute kidney injury (AKI) for a long time [21]. In addition to its status as an early predictor of AKI, recent studies have suggested that LCN2 could serve as a contributor to CKD [17, 22, 23]. Interestingly, positive correlations between circulating LCN2 and serum calcium or aortic root calcium volume were observed in CKD patients, indicating that LCN2 levels is not only a biomarker for kidney injury but also a possible participant in the pathogenesis of CKD-VC [16, 24]. However, there is no experimental evidence confirming the role of LCN2 in CKD-VC. This study extends the above findings by demonstrating the role of LCN2 in CKD-VC from multiple perspectives via a clinical study and in vitro and in vivo experimental models. Through our clinical study, we discovered that increased serum LCN2 expression was associated with severe aortic calcification in CKD patients. Our study also provided direct evidence of a positive correlation between LCN2 expression and CKD-VC presence in the radial arteries of HD patients. Furthermore, we found that LCN2KO mice were less vulnerable to AP diet-induced CKD-VC than were WT mice. These data indicated the protective role of LCN2 inhibition in CKD-VC. In our present study, we also confirmed the protective effect of LCN2 inhibition on vascular phenotypic remodelling. We observed that genetic inhibition of LCN2 in VSMCs also inhibited HP-induced calcium deposition and phenotypic switching. Similarly, LCN2 was reported to drive the phenotypic switching of VSMCs in atherosclerotic calcified plaques [14]. Intriguingly, the pharmacological blockade of LCN2 has been shown to prevent osteogenic remodelling of valve interstitial cells (VICs) in the calcific aortic valve [15]. However, the intracellular silencing of LCN2 in patient-derived calcifying VICs was found to exacerbate calcium deposition and osteogenic phenotype transition after HP stimulation. The discrepancy between the results might be explained by differences in disease backgrounds. These paradoxical effects were not observed under CKD-VC conditions, suggesting that CKD-VC is an independent pathological process compared to other calcific diseases. Moreover, the VICs extracted from the severely calcified valves used in that study differed from the VSMCs we used, suggesting that the roles of LCN2 may vary at different stages of calcification. Our results from recombinant protein intervention experiments also consistently demonstrated that LCN2 exacerbated calcium deposition in VSMCs under HP conditions. Despite the overlapping bone-vascular axis in the pathogenesis of CKD-VC, the role of LCN2 in regulating the osteogenesis of pre-osteoblasts in bone diseases is a matter of debate [25–27]. Thus, the regulatory role of LCN2 in renal osteodystrophy is an interesting issue and further investigation is needed.

Mounting evidence supports a critical role for ferroptosis in VSMCs calcification and phenotypic switching [4–7]. Ferroptosis is a distinctive type of cell death characterised by the overproduction of lipid peroxidation products derived from iron metabolism [28]. Previous studies have focused mainly on atherosclerotic calcification and hyperlipidemia-associated VC due to lipid metabolism disturbances. However, the effect of ferroptosis on CKD-VC is still poorly understood. To date, only one publication has addressed this issue [7]. Iron metabolism is strongly disrupted in CKD patients [29]. Thus, it is essential to determine whether ferroptosis occurs in the arteries of patients with CKD-VC. Regrettably, the consistency between experimental findings from previous studies and findings derived from CKD-VC patients has not been explored. In this study, we observed more iron deposits in the radial arteries of HD patients with VC than in those without VC, confirming the role of ferroptosis in CKD-VC.

As an iron-trafficking protein, LCN2 has been reported to play a critical role in regulating ferroptosis [30]. However, the role of LCN2 in ferroptosis under different pathophysiological conditions remains a matter of debate. In metabolic and immune-related diseases, LCN2 expression is robustly upregulated, resulting in severe ferroptosis [10, 31]. In line with these findings, our results revealed that elevated LCN2 levels contributed to the ferroptosis of VSMCs in CKD-VC and that the downregulation of LCN2 expression reduced free iron and lipid oxidation, suggesting that the regulatory effect of LCN2 on CKD-VC occurs through the regulation of VSMCs ferroptosis. Conversely, LCN2 was reported to be a repressor of tumorigenic ferroptosis [32, 33]. Thus, the regulatory roles of LCN2 in ferroptosis-related diseases should be investigated in more detail. Unlike other ferroptosis-related diseases, iron metabolism disorders in CKD are complex. Renal iron overload and circulating iron deficiency are contradictory and coexist in both CKD patients and animal models [34, 35]. In our current study, we found that LCN2 deficiency ameliorated serum iron deficiency (Supplementary Fig. 3B) and reduced aortic tissue ferroptosis in the CKD-VC mouse model. In line with our observations, a positive association was observed between upregulated LCN2 expression and iron deficiency anaemia in HD patients [36]. Additionally, the deletion of LCN2 has been shown to lead to increased circulating iron levels in a Col4a3KO-induced CKD mouse model [37]. These findings suggest that LCN2 is a promising target for the treatment of dysregulated iron metabolism and renal anaemia in CKD patients; however, further investigation is needed.

Although LCN2 has been reported to induce ferroptosis in various diseases, the mechanisms underlying LCN2-induced ferroptosis remain unknown. Ferroptosis is a type of autophagy-dependent cell death. Ferritinophagy, a novel selective form of autophagy, largely contributes to the ferroptosis process [38]. In ferritinophagy, NCOA4, as a cargo receptor, delivers ferritin to lysosomes and ferritin is then degraded to release labile iron, which subsequently causes excessive lipid peroxidation to induce cell death. Ferritinophagy-induced ferroptosis is involved in various cardiovascular diseases [39–41]. However, the role of NCOA4/FTH1-mediated ferritinophagy in CKD-VC has not been reported. Notably, multiple previous studies have also demonstrated that the induction of FTH1 prevents CKD-VC through its ferroxidase activity [42, 43] More recently, NCOA4 has been reported to be involved in the VSMCs senescence [44]. Chronic senescence of vascular wall VSMCs is also a critical factor in the pathogenesis of CKD-VC [45]. Therefore, we assumed that HP contributes to VSMCs ferroptosis through ferritinophagy. In this study, we found that HP conditions induced ferritinophagy activation in VSMCs, as indicated by the inverse relationship between NCOA4 and FTH1 expression in the arteries of CKD patients and HP-stimulated VSMCs. NCOA4 knockdown by siRNA significantly reduced calcium deposition in HP-stimulated VSMCs, indicating that ferritinophagy is involved in CKD-VC. Furthermore, the inhibition of LCN2 significantly reversed the upregulation of NCOA4 expression and downregulation of FTH1 expression. HP-induced ferritinophagy in VSMCs was further augmented by recombinant LCN2 treatment under HP stimulation, indicating a specific regulatory role of LCN2 in NCOA4/FTH1-mediated ferritinophagy under CKD-VC conditions. Taken together, our findings revealed an unknown link between NCOA4/FTH1-mediated ferritinophagy and CKD-VC. Inhibiting LCN2-induced ferritinophagy may be a prospective therapeutic strategy for CKD-VC, but further studies are needed to demonstrate the clinical potential of this approach in patients with CKD-VC.

Our study has several limitations. First, the direct regulatory relationship between LCN2 and NCOA4 needs to be further validated. Second, in CKD, there are numerous reasons for the increase of LCN2 levels and the main production site remains unclear. Future studies of transgenic mice with VSMCs-specific LCN2 knockout will be essential to clarify the role of LCN2 in CKD-VC.

In summary, we investigated the role of LCN2 in the pathogenesis of CKD-VC. Our results showed that the inhibition of LCN2 may prevent the progression of CKD-VC by inhibiting HP-induced ferroptosis in VSMCs. Furthermore, the inhibition of LCN2 alleviated HP-induced VSMCs ferroptosis by inhibiting NCOA4/FTH1-mediated ferritinophagy. It is conceivable that strategies targeting LCN2 will have therapeutic potential in patients with CKD-VC.

## Materials and methods

### CKD patient samples

Peripheral venous blood samples were collected from patients with CKD and 20 healthy people undergoing physical examinations at Zhongda Hospital of Southeast University, China, from October 2020 to December 2023. The inclusion criteria were (a) age ≥ 18 years; (b) the availability of abdominal radiographs or chest CT images; and (c) the presence of an abnormality in kidney structure or function persisting for more than 3 months. A total of 352 patients were included in this study. Individuals meeting any of the following criteria were excluded: (a) individuals who participated in other interventional studies; (b) individuals who were pregnant or lactating; (c) individuals who had active inflammation or high serum C-reactive protein levels; and (d) individuals who had malignancies, cirrhosis, heart failure or other life-threatening diseases.

The sample size for our cross-sectional study was calculated based on the formula for estimating the sample size in clinical studies. The sample size formula used is: $n=\frac{{Z}^{2}\cdot P\cdot Q}{{d}^{2}}$. The expected prevalence rate (*P*) was derived from a previous large-scale clinical study [46], which reported a vascular calcification (VC) prevalence of 77.1% in patients with stage 5 CKD. Therefore, *P* = 0.771 and *Q* = 1 *−* *P* = 0.229. *Z* is the standard normal deviate at a 95% confidence level (*Z* = 1.96) and *d* is the margin of error, set at 10% of the expected prevalence (*d* = 0.1$\cdot$ 0.771 = 0.0771). Substituting these values into the formula gives: n = 118.80. Therefore, to ensure adequate statistical power and reliability, we determined that a minimum of 118 patients was required for our study.

A total of 160 CKD patients were ultimately recruited for this clinical study. CKD patients were divided into non-VC group and VC group on the basis of their calcification score from abdominal radiography or chest CT. Clinical and biochemical parameters were collected from the electronic medical records at the hospital. Serum samples were obtained by centrifuging peripheral venous blood samples at 3000 rpm for 15 min at 4 °C; the samples were stored at −80 °C until use. To investigate the association between LCN2 levels and the presence of CKD-VC, serum LCN2 levels were measured with an enzyme-linked immunosorbent assay (ELISA) kit (catalogue no. E-EL-H6127; Elabscience, Wuhan, China). The Kauppila semiquantitative scoring method was used to evaluate the abdominal radiographs of CKD patients and to calculate the AAC score [47]. Patients with CKD-VC were divided into the following groups on the basis of AAC score: the no or mild abdominal calcification (AAC score ≤ 4), moderate abdominal calcification (AAC score between 5 and 15) and severe abdominal calcification (AAC score ≥ 16) groups. The aortic arch is more susceptible to calcification than other regions of the thoracic aorta. Therefore, quantitative assessment of aortic arch calcification was conducted by using mediastinal window settings on chest CT scans and a slice thickness of 1.25 mm. The calcium volume was precisely quantified by segmenting areas with a density threshold of ≥130 Hounsfield units (HU) from the volume-rendered images via semiautomatic software (Syngo.Via, Siemens Healthcare GmbH, Erlangen, Germany) for image analysis. All the calcium volume scores for the images were blindly quantified by 2 independent investigators.

A 3–6 mm segment of the radial artery was removed from HD patients who underwent an arterial venous fistula operation. The degree of calcification in the radial artery was determined via Von Kossa staining. Specifically, arterial sections were treated with 5% silver nitrate, exposed to ultraviolet light for 30 min, rinsed and incubated with 5% sodium thiosulfate. The calcified spots were stained dark brown. The areas with positive Von Kossa staining were analysed with ImageJ software (National Institutes of Health). To further investigate the association between LCN2 expression and the presence of CKD-VC, the expression of LCN2 in radial arteries was examined by immunofluorescence staining. Radial arterial sections were incubated with a primary antibody against LCN2 (1:100; Santa Cruz, sc-518095) at 4 °C overnight and then incubated with Alexa Fluor 594-conjugated goat anti-mouse IgG (1:200; Jackson) for 2 h at room temperature. Nuclei were stained with DAPI (Beyotime Biotechnology, China). The fluorescence images were scanned through a panoramic 250 Flash II slide scanner (3DHISTECH, Budapest, Hungary) and viewed with panoramic Viewer Software (3DHISTECH, Budapest, Hungary). Semiquantitative analyses of calcification and the mean fluorescence intensity of LCN2 were performed with ImageJ software (ImageJ, National Institutes of Health).

### Animal studies

All animal experiments were performed in accordance with standard guidelines for the care and use of laboratory animals. The animals were raised at the Laboratory Animal Centre of Southeast University and maintained in a specific pathogen-free environment (12-h/12-h light/dark cycle, 23 ± 2 °C, humidity 50 ± 10%) with free access to food and purified water.

The sample size for the animal study was set to a minimum of 5 subjects per group. This decision aligns with standard practices in the field, which recommend a minimum sample size to ensure the validity and reliability of the results. A sample size of at least 5 animals per group accounts for biological variability and ensures that the study can identify meaningful differences between groups, even in the absence of formal statistical power calculations. This approach is consistent with ethical guidelines, aiming to minimise the number of animals used while still ensuring the scientific validity and reproducibility of the results.

The CKD-VC rat model was established by performing 5/6 nephrectomy and providing a HP diet as described in our previous study [48]. Briefly, after 2 weeks of acclimation feeding to gain body weight, male Sprague-Dawley (SD) rats (200–250 g) were anaesthetised and 5/6 nephrectomy was performed in 2 steps to induce CKD. In the first week, two-thirds of the right kidney was removed and in the second week, the total left kidney was removed. After 1 week of recovery, the rats subjected to 5/6 nephrectomy (n = 15) were fed an HP diet (1.8% phosphate) (Xietong Pharmaceutical Bioengineering Co. Ltd. Nanjing, China) for the subsequent 12 weeks. Control rats (n = 6) underwent a sham operation and were fed a normal diet.

For the AP diet-induced CKD-VC mouse model, male C57BL/6J mice aged 8 weeks and weighing 22 to 25 grams were randomly assigned to the control group or experimental group. The mice in the control group (n = 6) were fed standard pellet chow (normal diet, ND) without the addition of adenine or phosphorus (Xietong Pharmaceutical Bioengineering Co., Ltd., Nanjing, China), and the mice in the CKD-VC model group (n = 14) were fed special chow containing 0.2% adenine (adenine, A8626, Sigma‒Aldrich, Saint Louis, USA) for 4 weeks to induce CKD and then fed a compound adenine and high-phosphorus diet containing 0.2% adenine and 1.8% phosphorus (Xietong Pharmaceutical Bioengineering Co., Ltd., Nanjing, China) for the subsequent 10 weeks. The details of the CKD-VC mouse model establishment protocol were described in the previous study of our laboratory [49]. After 14 weeks of AP diet induction, all mice were sacrificed and blood and urine were collected.

Male LCN2 KO and WT mice on a C57BL/6J genetic background were obtained from GemPharmatech Co., Ltd. (C57BL/6JGpt-LCN2em5Cd5686/Gpt, strain no. T014584). CRISPR/Cas9 technology was used to delete a 5686-bp sequence in exons of the LCN2 gene. Tail DNA was used to confirm gene targeting at the age of 3 weeks. The genetic identification is detailed in the Supplementary Fig. 4. The modification of the genotype had no discernible effect on initial body weight, health status, or immunological status. After 2 weeks of acclimation, 8-week-old male LCN2 KO and WT mice were randomly divided into normal diet group (n = 6 per group) and AP diet group (n = 14 per group).

For VSMCs-specific LCN2 overexpression, WT C57BL/6J mice were injected via the lateral tail vein with recombinant adeno-associated virus serotype 9 gene (AAV9) transfer vectors bearing a VSMCs-specific promoter combination (SM22α promoter) with a mouse LCN2-overexpression sequence. AAV9 was generated by Brain-VTA (Wuhan, China). The mice were injected via the lateral tail vein with either 100 µl of AAV9-LCN2 (rAAV-SM22a-Lcn2-P2A-mCherry-WPRE-hGH pA, 1 × 10^11^ vg/ml) or the AAV9-negative control (AAV-NC) (rAAV-SM22a-mCherry-WPRE-hGH pA, 1 × 10^11^ vg/ml). The identification of AAV9-mediated VSMCs targeting is detailed in Supplementary Fig. 5. Two weeks after tail vein injection, the mice in the AAV9-NC injection group and the AAV9-LCN2 injection group were randomly divided into the normal diet group (n = 8 per group) and AP diet group (n = 12 per group).

### Blood biochemical analyses

Rat serum levels of creatinine (Scr), blood urea nitrogen (BUN), calcium, phosphorus and iron were measured with commercial assay kits (catalogue no. C011-2-1, C013-2-1, C004-1, C006-1-1; Nanjing Jiancheng Bioengineering Institute, Nanjing, China)

Mouse serum levels of creatinine (Scr), blood urea nitrogen (BUN), calcium, phosphorus and iron were measured with an automatic biochemistry analyser (Chemray, Ledu Life Technology, Shenzhen, China).

### Cell culture and VSMCs calcification induction

Mouse VSMCs were purchased from CellCook (catalogue no. CC9011) and cultured in high glucose DMEM (4.5 g/L glucose) (catalogue no. 319-006-CL; Wisent Corporation, Nanjing, China) supplemented with 10% FBS (catalogue no. 10099141 C, Gibco) at 37 °C and 5% CO_2_ in a humidified saturated incubator. Cells up to passages 5–8 were used for experiments.

In our in vitro experiments, the sample size for cell studies was set to a minimum of 3 independent replicates. This choice was made to ensure the reliability and consistency of our results. By using at least 3 replicates, we can effectively account for biological variability and confirm the reproducibility of our findings. This approach allows us to draw meaningful and robust conclusions from our data.

To induce calcification, VSMCs were incubated in calcifying medium (HP) containing high-glucose DMEM supplemented with 0.5% FBS and sodium phosphate (catalogue no. 94046, Sigma‒Aldrich) at a final concentration of 3.0 mmol/L in an incubator containing 5% CO_2_ for 5 days. The culture medium was changed every 2 days. The control VSMCs were incubated in growth medium containing high-glucose DMEM supplemented with 0.5% FBS but without sodium phosphate, and the medium was also changed every 2 days.

VSMCs were incubated with recombinant murine LCN2 (rmLCN2, catalogue no. HP17, Novoprotein, Shanghai, China) under CTL and HP conditions (final concentration, 1000 ng/ml) for 5 days. VSMCs were incubated with LCN2 inhibitor ZINC00784494 (catalogue no. HY-148364, MedChemExpress, New Jersey, USA) under HP conditions (final concentration, 0.5 µmol/L /ml) for 5 days. For siRNA transfection, VSMCs were seeded at 5 × 10^5^ cells/well in 6-well plates. When VSMCs reached 50% confluence, they were transfected with a specific siRNA (final concentration, 10 nmol/L) via Lipofectamine 2000 (Invitrogen) according to the manufacturer’s instructions. Eight hours after transfection, the serum-free high-glucose DMEM was replaced with calcification-induction medium. The specific sequences of the siRNAs used are listed in the Supplementary Table 2. To overexpress LCN2, full-length mouse LCN2 cDNA was cloned and inserted into a lentiviral vector (Lv-LCN2) and an empty lentiviral vector (Lv-NC) was used as the control (GeneChem Co., Ltd., Shanghai, China); VSMCs were infected with lentivirus according to the manufacturer’s instructions [multiplicity of infection (MOI = 50)]. Stable colonies were selected by treatment with 5 μg/mL puromycin (Beyotime, China). The overexpression and silencing efficiencies were examined via qRT‒PCR and Western blot.

### Immunofluorescence staining

Radial arteries from HD patients and mouse aortas were fixed in 4% paraformaldehyde and embedded in paraffin. Paraffin-embedded vascular sections (6 μm) were first placed in an oven at 60 °C for 2 h. Then, the sections were deparaffinized in xylene and rehydrated through a graded series of ethanol. Antigen retrieval was performed by microwave irradiation in citrate buffer at pH 6.0. The protocol included initial heating at medium power for 8 min, followed by a cooling period of 8 min, followed by an additional 7 min of heating at medium-low power. For immunofluorescence staining, the sections were incubated with 5% bovine serum albumin (BSA) in phosphate-buffered saline (PBS) for 1 h at room temperature to reduce nonspecific background staining. The sections were then incubated with primary antibodies at 4 °C overnight, which was followed by incubation with Alexa Fluor (Jackson ImmunoResearch) secondary antibodies for 2 h at room temperature. Nuclei were stained with DAPI (Beyotime Biotechnology, China). Fluorescence images were captured with an Olympus FV3000 fluorescence confocal microscope or Panoramic 250 Flash II slide scanner (3DHISTECH, Budapest, Hungary) and viewed with Panoramic Viewer Software (3DHISTECH, Budapest, Hungary). Semiquantitative analyses of calcification and fluorescence intensity were performed with ImageJ software (ImageJ, National Institutes of Health).

### Immunohistochemistry

Vascular sections (6 μm) were dewaxed and rehydrated. Heat-mediated antigen retrieval was performed using citrate buffer at pH 6.0. Endogenous peroxidase activity was blocked with 0.3% H_2_O_2_ at room temperature for 10 min, followed by blocking with 10% goat serum for 1 h at room temperature. The sections were incubated with primary antibodies overnight at 4 °C. After three washes with PBS, the tissue sections were processed with the Super Horseradish Peroxidase (HRP) Mouse/Rabbit Immunohistochemistry (IHC) Kit (MXB Biotechnologies, China) according to the manufacturer’s instructions. The visualisation of peroxidase conjugates was facilitated through the use of a diaminobenzidine (DAB) kit (MXB Biotechnologies, China). Thereafter, the sections were counterstained with haematoxylin (Servicebio, China). Photomicrographs were acquired with an Olympus optical microscope, and subsequent analysis was performed with ImageJ software (National Institutes of Health).

### Aortic calcification quantification

Aortic calcification was detected by Von Kossa staining. Aortic sections were treated with 5% silver nitrate, exposed to ultraviolet light for 30 min, rinsed and incubated with 5% sodium thiosulfate. The calcified spots were stained dark brown. Each section was examined and scored as follows by more than two examiners: 1 = no staining; 2 = very little staining; 3 = sparse staining; 4 = uniform staining; and 5 = strong and widespread staining.

Aortic calcium deposition was quantified with an o-cresolphthalein complex using a commercial Ca assay kit (catalogue no. S1063, Beyotime, China) and following the manufacturer’s instructions. In brief, arterial tissues were homogenised and centrifuged, after which the supernatant was retained. Then, 150 μl of o-cresolphthalein solution was added to the supernatant, followed by mixing and incubation for 15 min at room temperature. The absorbance at 575 nm was measured with a microplate reader. The protein concentration was measured with a bicinchoninic acid (BCA) protein assay kit, and the relative calcium concentration was normalised to the protein concentration.

For alizarin red staining of whole-mount aortic segments, mouse aortic tissues were fixed in 95% ethanol for 24 h and then stained with 0.003% alizarin red solution in 1% potassium hydroxide for 30 h. Aortic tissues were then rinsed with 2% potassium hydroxide and photographed with a digital camera.

### In vitro VSMCs calcification quantification

The presence of in vitro calcification was determined by alizarin red staining. After the cells were incubated with calcifying medium, the culture medium was discarded and the treated VSMCs were washed with PBS three times. Then, VSMCs were fixed in 4% paraformaldehyde (Servicebio, Wuhan, China) for 20 min. After being washed three times with PBS, the fixed VSMCs were stained with 2% alizarin red S staining solution (pH 4.2; Servicebio, Wuhan, China) for ~10 min. After the samples were washed three times with double distilled water, images were acquired with an inverted microscope. To quantify alizarin red staining, 100 mmol/L hexadecylpyridinium chloride (catalogue no. C9002, Sigma) was used for decolorisation and the optical density (OD) at a wavelength of 560 nm was measured with hexadecylpyridinium chloride solution as blank.

To quantify the cellular calcium concentration, the VSMCs were washed three times with PBS after treatment and decalcified with 0.6 mol/L HCl (catalogue no. 30721, Sigma) for 24 h at 4 °C. After 24 h of incubation, the hydrogen chloride supernatant was collected for determination of calcium concentration with a calcium assay kit (catalogue no. S1063, Beyotime, China). Following decalcification, the cells were washed twice with PBS and solubilized with a solution of 0.1 mol/L NaOH (catalogue no. S8045, Sigma) and 0.1% sodium dodecyl sulphate (catalogue no. ST627, Beyotime, China), and protein concentration of the samples was measured via a BCA protein assay kit (catalogue no. KGB2101-100, KeyGen BioTECH, China). The calcium concentration of the VSMCs was normalised to the protein concentration and is expressed as µg/mg protein.

### Cell viability assay

The viability of the VSMCs was quantitatively assessed with the Cell Counting Kit-8 (CCK-8) assay (catalogue no. C0038, Beyotime, China), which was conducted in strict accordance with the protocol provided by the manufacturer. VSMCs were plated at a density of 5 × 10^3^ cells per well in 96-well microtiter plates. After seeding, the cells were exposed to calcification-inducing medium supplemented with or without the ferroptosis inhibitor Fer-1 (catalogue no. 347174-05-4, Sigma) for 5 days. To conduct the viability assay, 10 μl of CCK-8 solution was added to each well, followed by incubation at 37 °C for 2 h. After incubation, the optical density of each well was measured at a wavelength of 450 nm with a spectrophotometric microplate reader.

### Intracellular total and lipid ROS assays

The total intracellular ROS levels in the VSMCs were measured via the fluorescent probe 2’,7’ -dichlorofluorescin diacetate (DCFDA; also known as H2DCFDA, DCFH-DA and DCFH; catalogue no. ab113851, Abcam). DCFDA stained images were obtained with an Olympus laser confocal microscope with excitation/emission at 485/535 nm. The total intracellular total ROS levels in HP-treated VSMCs were shown in Supplementary Fig. 6A, B.

Lipid ROS levels in VSMCs were measured via the fluorescent probe C11-BODIPY^581/591^ (catalogue no. D3861; Invitrogen, USA). Briefly, VSMCs were incubated with C11-BODIPY^581/591^ (5 μM) in light-protected confocal dishes for 30 min and then washed three times with PBS. Finally, the nuclei were stained with Hoechst 33342 (catalogue no. R37165, Invitrogen, USA). After live-cell C11-BODIPY^581/591^ staining, the VSMCs were washed with PBS. Images were acquired under an FV3000 fluorescence microscope (Olympus, Tokyo, Japan). Analysis of C11 BODIPY ^581/591^ was performed by measuring the intensity of fluorescence with ImageJ software (National Institutes of Health). The lipid ROS levels in HP-treated VSMCs are shown in Supplementary Fig. 6C.

### Detection of lipid peroxidation in VSMCs

Lipid peroxidation was evaluated by measuring the GSH and MDA content. A GSH detection kit (catalogue no. S0053; Beyotime, China) was used to measure the total glutathione concentration in the calcifying medium of the treated VSMCs. The experiment was conducted in strict accordance with the instructions provided. The MDA content in calcified aortas was assessed with an assay kit (catalogue no. S0131S, Beyotime, Shanghai, China) according to the manufacturer’s instructions. In brief, aortic tissues were homogenised and centrifuged, after which the supernatant was retained. Thiobarbituric acid (TBA) solution was then added to the supernatant and reacted in an acidic and high-temperature environment for 15 min. After the reaction, the supernatant was obtained by centrifugation and the absorbance was evaluated at 532 nm with a microplate reader (Thermo Fisher Scientific, USA).

### Determination of iron concentration

The intracellular labile ferrous iron pool (LIP) in the VSMCs was measured with the fluorescent probe FerroOrange (catalogue no. F374; Dojindo, Japan). After the indicated treatments, the supernatant was discarded and the cells in the confocal dishes were washed three times with PBS. FerroOrange (5 μmol/L, Dojindo, Japan) working solution was subsequently added to the cells in the confocal dishes, which were subsequently incubated in an incubator with 5% CO_2_ and 95% air at 37 °C for 30 min and photographed under a confocal microscope (FV3000, Olympus, Japan) or with a multifunctional fluorescence microplate detection system (Thermo Fisher Scientific, USA). Total intracellular iron levels in VSMCs were analysed with an iron assay kit (Abcam, ab83366) following the manufacturer’s instructions.

### Iron staining

A prussian blue iron staining kit (G1029, Servicebio, Wuhan, China) was used to determine iron accumulation in the radial arteries and aortas according to the manufacturer’s instructions. After dewaxing and rehydration, the vascular sections were immersed in staining solution for 1 h, rinsed with distilled water and subsequently stained with nuclear fast red solution for 5 min. Finally, the sections were sequentially dehydrated with a gradient series of ethanol, cleared with xylene and then mounted with neutral mounting medium. The nuclei were stained red, and blue staining indicated iron deposition. Images were acquired via an Olympus optical microscope, and subsequent analysis of the positively stained areas was performed with ImageJ software (National Institutes of Health).

### Transmission electron microscopy

Aortic tissues and cells were fixed in 2.5% glutaraldehyde (obtained from Pinuofei, Wuhan) at a controlled temperature of 4 °C. The preparation and analysis of samples for TEM were performed by Wuhan Pinuofei Biological Technology Co., Ltd. Following initial embedding in a 1% agarose solution, the samples were further fixed with 1% osmium tetroxide (OsO4) in 0.1 mol/L PBS (pH 7.4) for 2 h at ambient temperature. After fixation, a graded series of ethanol was used to dehydrate the samples, which were then infiltrated and embedded within a resin matrix. To induce polymerisation, the resin-embedded samples were incubated in an oven at 65 °C for a minimum of 48 h. Ultrathin sections ranging from 60 to 80 nm in size were obtained with an ultramicrotome (Leica) and transferred onto 150-mesh copper grids coated with a Formvar film. These sections were stained sequentially with uranyl acetate dissolved in absolute ethanol and lead citrate. Following an overnight air-drying period at room temperature, the ultrastructural details were visualised with a Hitachi HT7700 transmission electron microscope. For a detailed analysis of mitochondrial morphology within vascular VSMCs, images were recorded at a magnification of 10,000×.

### Co-immunoprecipitation (Co-IP)

Total cell lysates were harvested using IP lysis buffer (catalogue no. P0013, Beyotime, Shanghai, China) containing 1% PMSF. Protein concentrations were determined using the BCA assay. The supernatant was divided into three portions: one portion was stored at −80 °C as the Input group. The other two portions were incubated with protein A/G agarose for 1 h on a 4 °C shaker to remove non-specific binding. Subsequently, these two portions were incubated overnight with either primary antibody or control IgG on a 4 °C shaker. The immune complexes were then pulled down with protein A/G agarose for 5 h on a 4 °C shaker. After centrifugation, the microbeads were collected and proteins were eluted to prepare protein samples, which were then analysed by Western blot analysis.

### Western blot analysis

Vascular specimens and VSMCs were lysed with radioimmunoprecipitation assay (RIPA) buffer (sourced from Keygen Biotech, China) containing the serine protease inhibitor phenylmethanesulfonyl fluoride (PMSF) and phosphatase inhibitors (also provided by Keygen Biotech, China). The concentration of proteins within the lysates was determined using a BCA protein assay kit (acquired from Keygen Biotech, China). Aliquots containing equivalent amounts of protein were subsequently loaded onto sodium dodecyl sulphate‒polyacrylamide gel electrophoresis (SDS‒PAGE) gels, and the resolved proteins were then electrophoretically transferred onto an Immobilon-P polyvinylidene difluoride (PVDF) membrane (manufactured by Merck Millipore, USA). The membrane was subsequently blocked with a 5% BSA in preparation for the immunodetection process. Primary antibodies were applied, and the membranes were incubated at 4 °C overnight. This step was followed by a 1-h incubation with horseradish peroxidase (HRP)-conjugated secondary antibodies. Quantitative analysis of protein band intensities was performed with ImageJ software (National Institutes of Health), enabling the assessment of relative protein expression levels derived from the developed immunoblots. The antibodies used are summarised in Supplementary Table 3.

### Quantitative real-time polymerase chain reaction analysis

Total RNA was extracted from the samples by using total RNA extraction reagent (Vazyme, China). This RNA was subsequently reverse transcribed into complementary DNA (cDNA) via reverse transcription SuperMix (Vazyme, China). Quantitative analysis of messenger RNA (mRNA) levels was performed utilising SYBR Green qPCR Master Mix (Vazyme, China). Glyceraldehyde-3-phosphate dehydrogenase (GAPDH) was used as the endogenous control for normalisation. The relative quantification of gene expression was achieved via the 2^-ΔΔCT^ method. The sequences of the primers are listed in Supplementary Table 4.

### Statistical analysis

Categorical data are presented as frequencies and percentages. Continuous variables are expressed as the mean ± standard deviation (SD) for normally distributed variables and as the median (interquartile range [IQR]) for variables with a skewed distribution. For comparisons between 2 groups, significance was determined via Student’s t test or the nonparametric Mann‒Whitney U test. Variances between the groups were compared using the F-test, if variances were unequal, an unpaired t-test with Welch’s correction was applied. For comparisons among multiple groups, one-way analysis of variance (ANOVA) was performed, followed by post hoc Bonferroni’s correction or Dunnett’s test. The statistical significance of the correlations was assessed via Spearman correlation coefficient analysis. A two-tailed *p* < 0.05 was considered to indicate statistical significance. Statistical analyses were performed with GraphPad Prism v9.5.1 (San Diego, CA, USA) and SPSS 27.0.1 (SPSS Inc., Armonk, NY, USA).

## Supplementary information

Supplemental material

Original images of representative western blot images

aj-checklist

## Data Availability

No publicly available data or shared data are cited. All original data supporting the conclusions of the current study are available from the corresponding author upon request.
