# Supplementary material for: Lipocalin-2 promotes CKD vascular calcification by aggravating VSMCs ferroptosis through NCOA4/FTH1-mediated ferritinophagy
Source: Cell Death Dis. 2024 Nov 29;15(11):865. doi: 10.1038/s41419-024-07260-x (PMC11607329; doi:10.1038/s41419-024-07260-x)
Supplement: Supplementary file 1 — Supplemental material [file 41419_2024_7260_MOESM1_ESM.pdf]

Supplementary Table 1. Baseline characteristics of the 20 healthy people included in this study

	**Total (n=20)**
**Age, years**	48.21±12.42
**Sex**	
**Male**	11（55%）
**Female**	9（45%）
**BMI, kg/m²**	25.11±2.86
**Serum LCN2,ng/ml**	43.12(21.47-64.16)
**Hemoglobin, g/L**	150.05±14.53
**eGFR, mL/min/1.73m²**	100.60±16.11
**Urea, mmol/L**	5.00（4.30-7.00）
**Creatinine, mmol/L**	73.42±14.24
**Calcium, mmol/L**	2.32±0.09
**Phosphate, mmol/L**	1.12±0.12
**Total cholesterol, mmol/L**	4.79（4.57-5.34）
**Triglycerides, mmol/L**	1.41（1.01-2.23）
**HDL cholesterol, mmol/L**	1.26±0.40
**LDL cholesterol, mmol/L**	2.97±0.78
**Lipoprotein A, mg/L**	124.00（62.50-215.00）

Supplementary Table1. Baseline characteristics of the 20 healthy people included in this study. The values are expressed as mean ±SD, median (25% to 75% quartiles) and n (%) for normally distributed continuous variables, skewed distributed continuous variables and categorical variables, respectively. BMI, body mass index; LCN2, lipocalin-2; eGFR, estimated glomerular filtration rate; HDL-cholesterol, high-density lipoprotein cholesterol; LDL-cholesterol, low-density lipoprotein cholesterol; SD, standard deviation.

Supplementary Table 2. The sequences of siRNAs used in this study

Gene	sense (5'-3')	Anti-sensse(5'-3')
Negative control	UUCUUCGAACGUGUCACGUTT	ACGUGACACGUUCGGAGAATT
Lcn2-Mus-356	CCAGUUCACUCUGGGAAAUTT	AUUUCCCAGAGUGAACUGGTT
Ncoa4-Mus-150	GGGCUGAACAGCAAAUUAATT	UUAAUUUGCUGUUCAGCCCTT

Supplementary Table 2. The sequences of the siRNA used in this study. siRNA, small interfering RNA.

Supplementary Table 3. The antibodies used in this study

Antibody name	Catalog number	Application
GAPDH	Proteintech (60004-1-lg)	1:50000 for western blot
β-actin	Proteintech (66009-1-lg)	1:1000 for western blot
RUNX2	CST (12556)	1:1000 for western blot
BMP2	Abcam (ab214821)	1:1000 for western blot
Lipocalin-2	R&D (AF1757)	1:1000 for western blot<br>1:100 for IHC
Lipocalin-2	SANTA CRUZ (sc-518095)	1:100 for IF
Lipocalin-2	Affinity (DF6816)	1:1000 for western blot
Lipocalin-2	R&D (AF1857)	1:1000 for western blot<br>2μg per 500μg of total protein<br>(1mL of cell lysate) for immunoprecipitation
NCOA4	SANTA CRUZ (sc-373739)	1:1000 for western blot<br>2μg per 500μg of total protein<br>(1mL of cell lysate) for immunoprecipitation
Goat IgG	Beyotime (A7007)	2μg per 500μg of total protein<br>(1mL of cell lysate) for immunoprecipitation
Mouse IgG	SANTA CRUZ (sc-69786)	2μg per 500μg of total protein<br>(1mL of cell lysate) for immunoprecipitation
FTH1	SANTA CRUZ (sc-376594)	1:100 for IF
FTH1	Abcam (ab183781)	1:1000 for western blot
GPX4	Abcam (ab125066)	1:1000 for western blot
HRP-conjugated affinipure goat anti-rabbit IgG(H+L)	Proteintech (SA00001-2)	1:5000 for western blot
HRP-conjugated affinipure rabbit anti-goat IgG(H+L)	Proteintech (SA00001-4)	1:5000 for western blot
HRP-conjugated affinipure goat anti-mouse IgG(H+L)	Proteintech (SA00001-1)	1:5000 for western blot
FITC-AffiniPure Goat Anti-Mouse IgG（H+L）	Jackson ImmunoResearch (115-095-003)	1:200 for IF
594-AffiniPure Goat Anti-Mouse IgG（H+L）	Jackson ImmunoResearch (115-585-003)	1:200 for IF

Supplementary Table 4. The sequences of the primers used in this study

Gene	Forward primer (5'-3')	Reverse primer (5'-3')
mouse-PTGS2	CAGTATCAGAACCGCATTG	CAGGAGGATGGAGTTGTT
mouse-GPX4	AGTACAGGGGTTTCGTGTGC	CATGCAGATCGACTAGCTGAG
mouse-RUNX2	ATCCCCATCCATCCACTCCA	GGGGTGTAGGTAAAGGTGGC
mouse-OCN	GGTAGTGAACAGACTCCGGC	TTAAGCTCACACTGCTCCCG
mouse-SM22α	GGTCCATCCTACGGCATGAG	TGCTCCTGGGCTTTCTTCATA
mouse-α-SMA	GTACCACCATGTACCCAGGC	GCTGGAAGGTAGACAGCGAA
mouse-LCN2	GGCCAGTTCACTCTGGGAAA	TGGCGAACTGGTTGTAGTCC
rat-LCN2	GGAATATTCACAGCTACCCTC	TTGTTATCCTTGAGGCCCAG
rat-GAPDH	CCTTCCGTGTTCCTACCC	CCTGCTTCACCACCTTCTT
mouse-GAPDH	AGGTCGGTGTGAACGGATTTG	GGGGTCGTTGATGGCAACA
mouse- β-actin	GGCTGTATTCCCCTCCATCG	CCCAGTTGGTAACAATGCCATGT
rat- β-actin	TCACCAACTGGGACGATA	GAGGCATACAGGGACAACA

Supplementary Figure 1

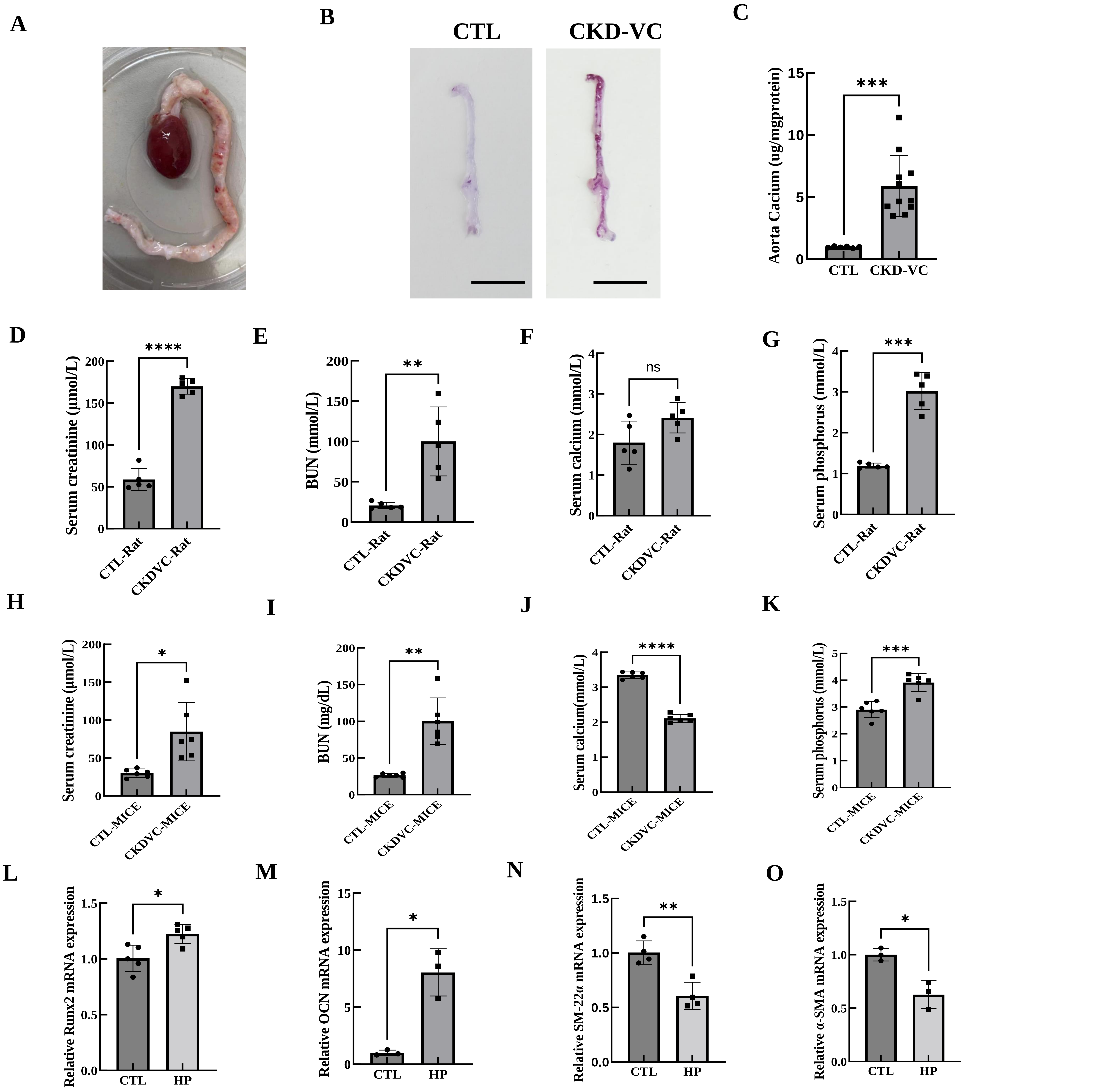

Supplementary Figure 1. Characteristics of the CKD-VC model rats and mice. A. General image of calcified aortas from CKD-VC rat. **B**. Alizarin red staining of whole aortas from CTL and CKD-VC mice. Scale bar: 1cm. **C**. The calcium concentrations of the aortas from each group were normalized to the related protein concentrations. (n=6 for CTL; n=11 for CKD-VC; $^{***}p < 0.001$ vs. CTL). Serum creatinine (**D**), BUN (**E**), serum calcium (**F**) and serum phosphorus (**G**) levels of CTL and CKD-VC rats. (n=5 per group, $^{**}p < 0.01$, $^{***}p < 0.001$ and $^{****}p < 0.0001$ vs. CTL rat). Serum creatinine(**H**), BUN (**I**), serum calcium (**J**) and serum phosphorus (**K**) levels in the CKD-VC model mice (n=6 per group, , $^{*}p<0.05$, $^{**}p < 0.01$, $^{***}p<0.001$ and $^{****}p<0.0001$ vs. CTL mice). **L**. Relative mRNA expressions of RUNX2 in VSMCs stimulated by HP (3.0 mM) (n=5 per group, $^{*}p<0.05$ vs. CTL). **M**. Relative mRNA expressions of OCN in VSMCs stimulated by HP (3.0 mM) (n=3 per group, $^{*}p<0.05$ vs. CTL). **N**. Relative mRNA expressions of SM-22α in VSMCs stimulated by HP (3.0 mM) (n=4 per group, $^{**}p<0.01$ vs. CTL). **O**. Relative mRNA expressions of α-SMA in VSMCs stimulated by HP (3.0 mM) (n=3 per group, $^{*}p<0.05$ vs. CTL). Data are presented as mean ± SD. RUNX2, runt-related transcription factor 2; OCN, osteocalcin; CKD-VC, chronic kidney disease-related vascular calcification; BUN, blood urea nitrogen; CTL, control.

Supplementary Figure 2

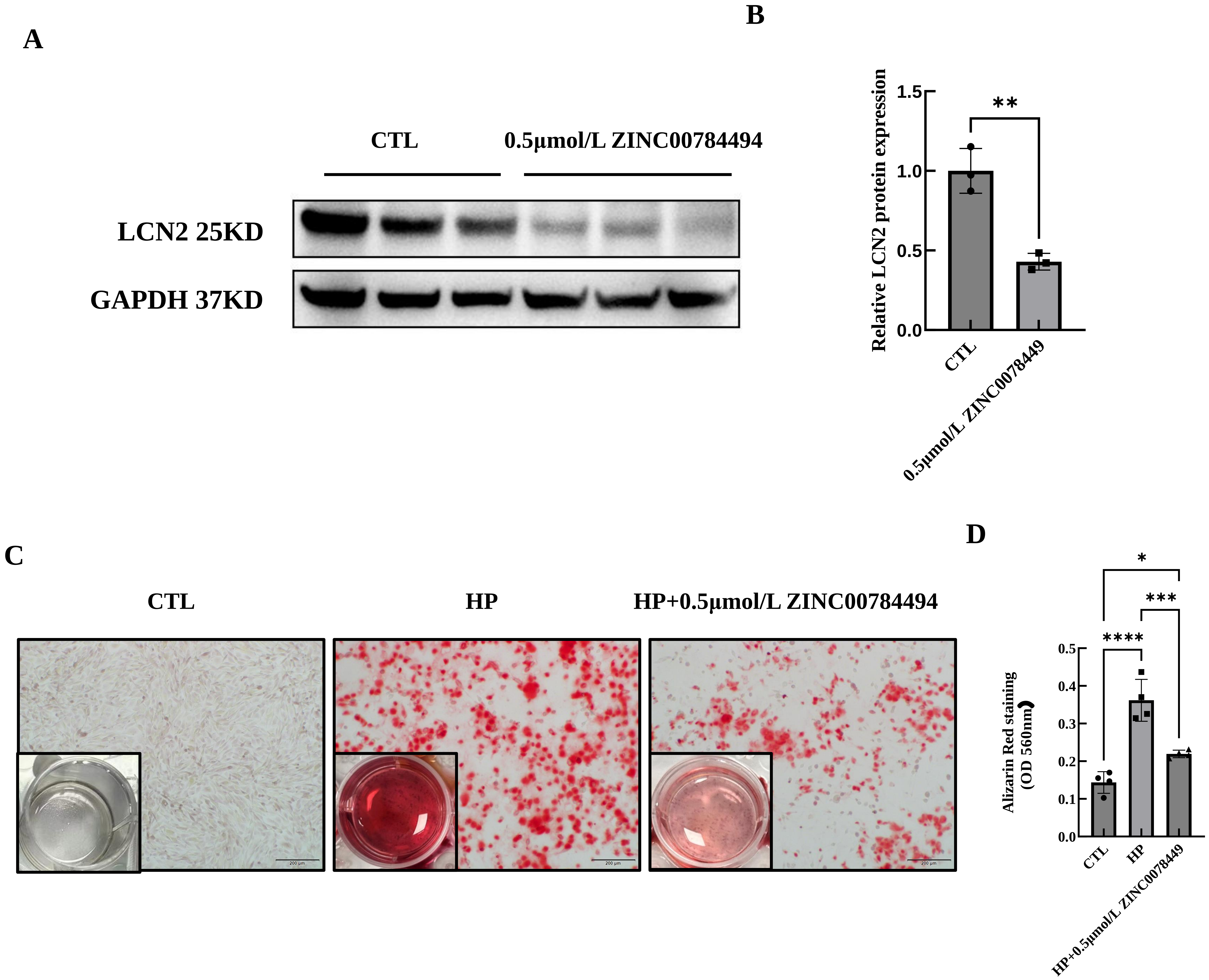

Supplementary Figure 2. LCN2 inhibition in vitro alleviates HP-induced VSMCs calcification. **A.** Representative Western blot images and quantification (**B**) of LCN2 protein expression in 0.5 µmol/L ZINC00784494-treated VSMCs (n=3 per group, $^{**}p$ <0.01 vs. CTL). **C.** Representative images of alizarin red staining of VSMCs treated with or without 0.5 µmol/L ZINC00784494 under HP conditions. Scale bar: 200 µm. **D.** Quantification of the alizarin red staining of VSMCs treated with or without 0.5 µmol/L ZINC00784494 under HP conditions using hexadecylpyridinium chloride at an OD of 560 nm. (n=4 per group, $^{*}p < 0.05$ and $^{****}p$ <0.0001 vs. CTL; $^{***}p$ <0.001 vs. HP). Data are presented as mean ± SD. CKD-VC, chronic kidney disease-related vascular calcification; VSMC, vascular smooth muscle cells; HP, high phosphate; ZINC00784494, LCN2 inhibitor.

Supplementary Figure 3

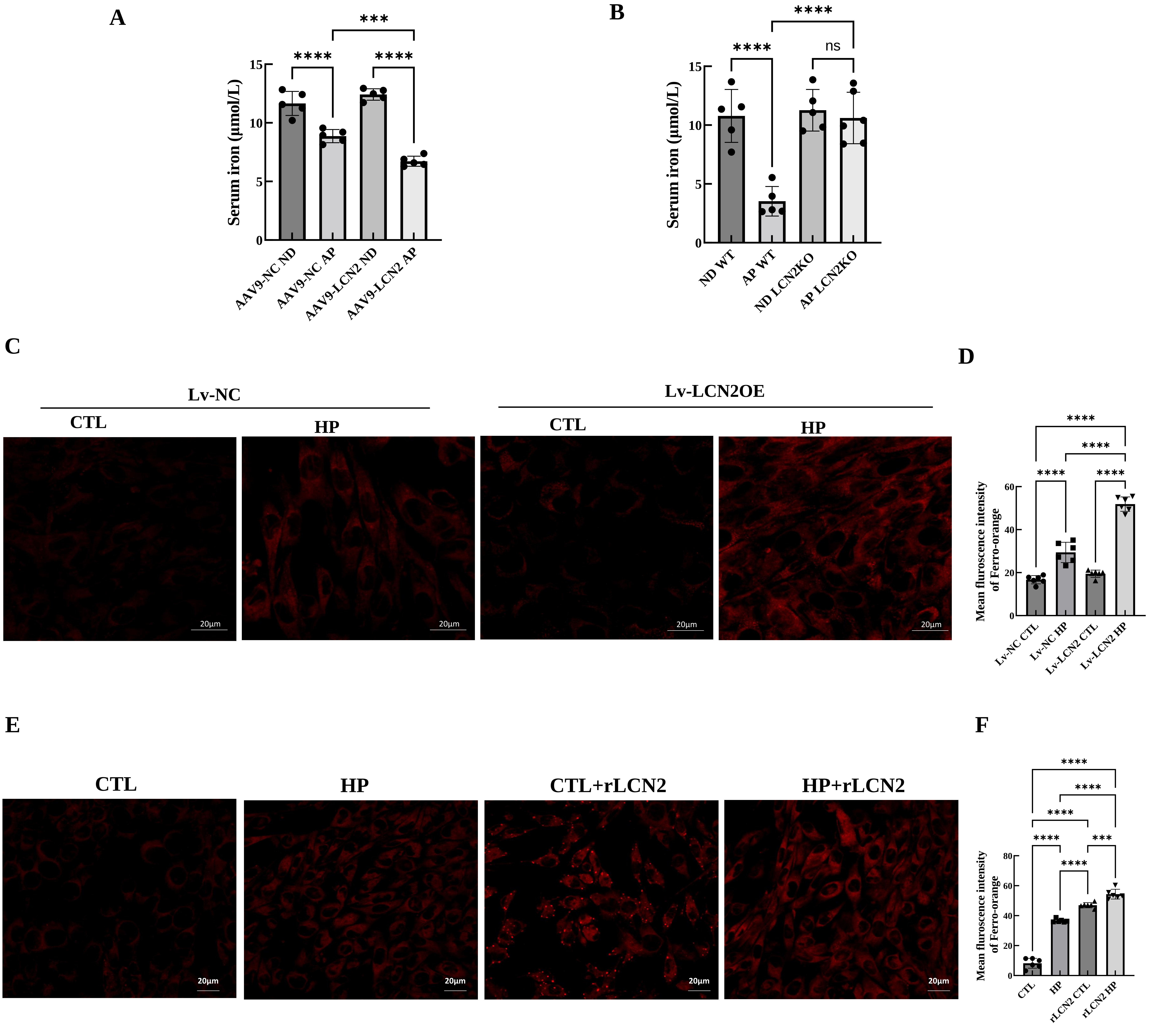

Supplementary Figure 3. LCN2 regulates iron metabolism in CKD-VC. A. Serum iron concentration of VSMCs-targeted LCN2-overexpressing mice and NC mice fed by the AP diet or ND. (n=5 per group). **B.** Serum iron concentration of the LCN2KO and WT mice fed by the AP diet or ND (n=5 per group). **C.** Representative fluorescence images and quantification (**D**) of the iron probe FerroOrange in LCN2-overexpressing VSMCs and NC VSMCs in the absence or presence of HP (n=6 per group). Scale bar:20μm. **E.** Representative fluorescence images and quantification (**F**) of the iron probe FerroOrange in recombinant LCN2-treated VSMCs in the absence or presence of HP (n=6 per group).Scale bar:20μm. Data are presented as mean ± SD. $^{*}p < 0.05$, $^{**}p < 0.01$, $^{***}p < 0.001$ and $^{****}p < 0.0001$. CKD-VC, chronic kidney disease-related vascular calcification; VSMC, vascular smooth muscle cells; AP, alanine and phosphate; NC, negative control; HP, high phosphate; LCN2KO, LCN2 knockout; WT, wild type.

Supplementary Figure 4

A

	Primer	**Sequence (5'-3')**	**Product size**
PCR①	GPS00001573-Lcn2-D5-5tF1	GTTGGATTCCAAAGGGCTCTCC	WT:6223bp -5686bp=537bp
	GPS00001573-Lcn2-D3-3tR1	CACTCACCACCCATTCAGTTGTC	
PCR②	JS13244-Lcn2-w t-tF1	TCAAGGTATTGGACACTTCCAGG	WT:434bp KO:0bp
	JS13244-Lcn2-w t-tR1	AACTGTGCAAGGTTGAGCAACAG	

B

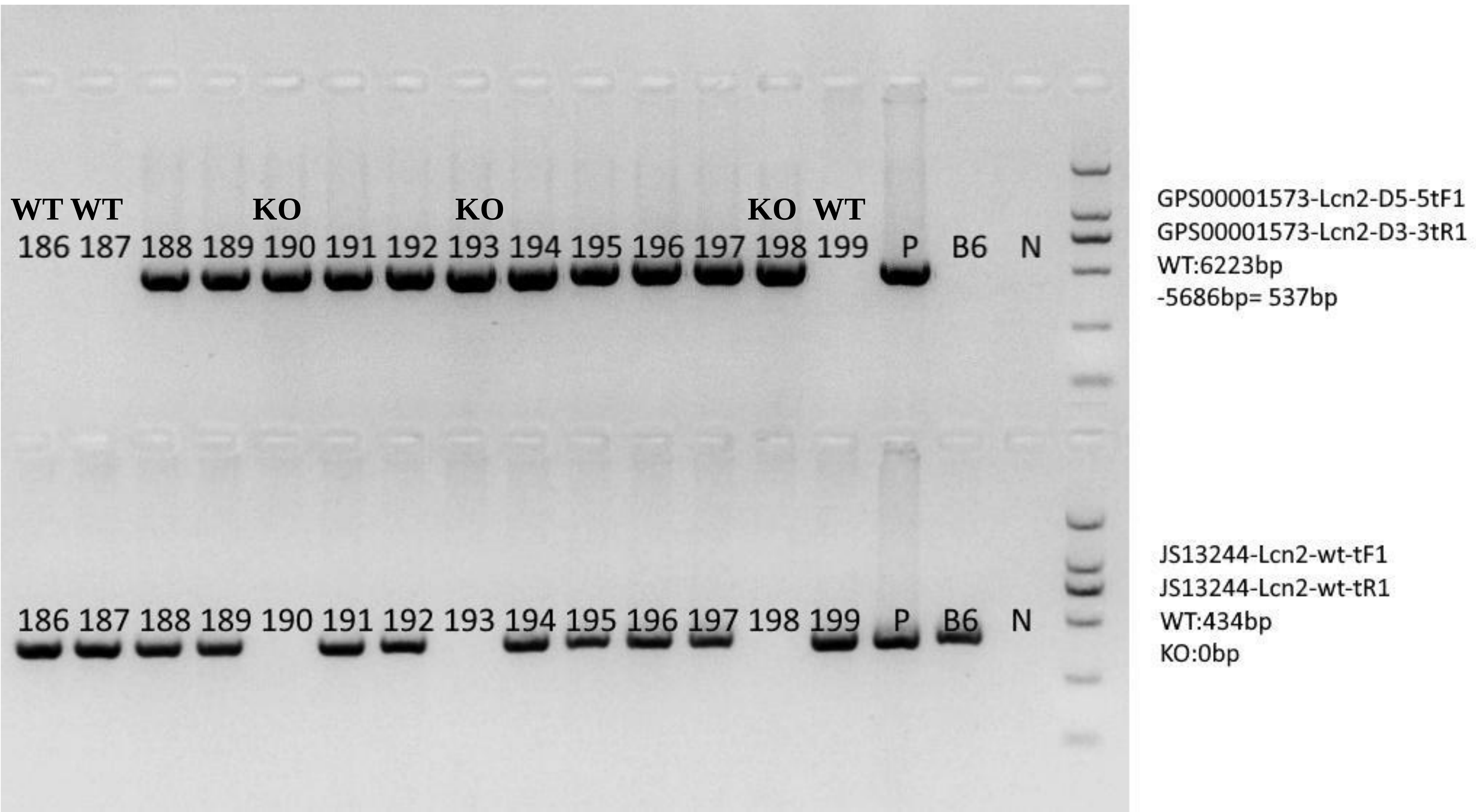

Supplementary Figure 4. Genetic identification of LCN2KO mice and WT mice. A. The sequences used in PCR for genetic identification. **B.** Representative images of genetic identification. KO, knockout; WT, wild type; PCR, polymerase chain reaction

Supplementary Figure 5

AAV9-mcherry-SM22α-NC

AAV9-mcherry-SM22α-LCN2

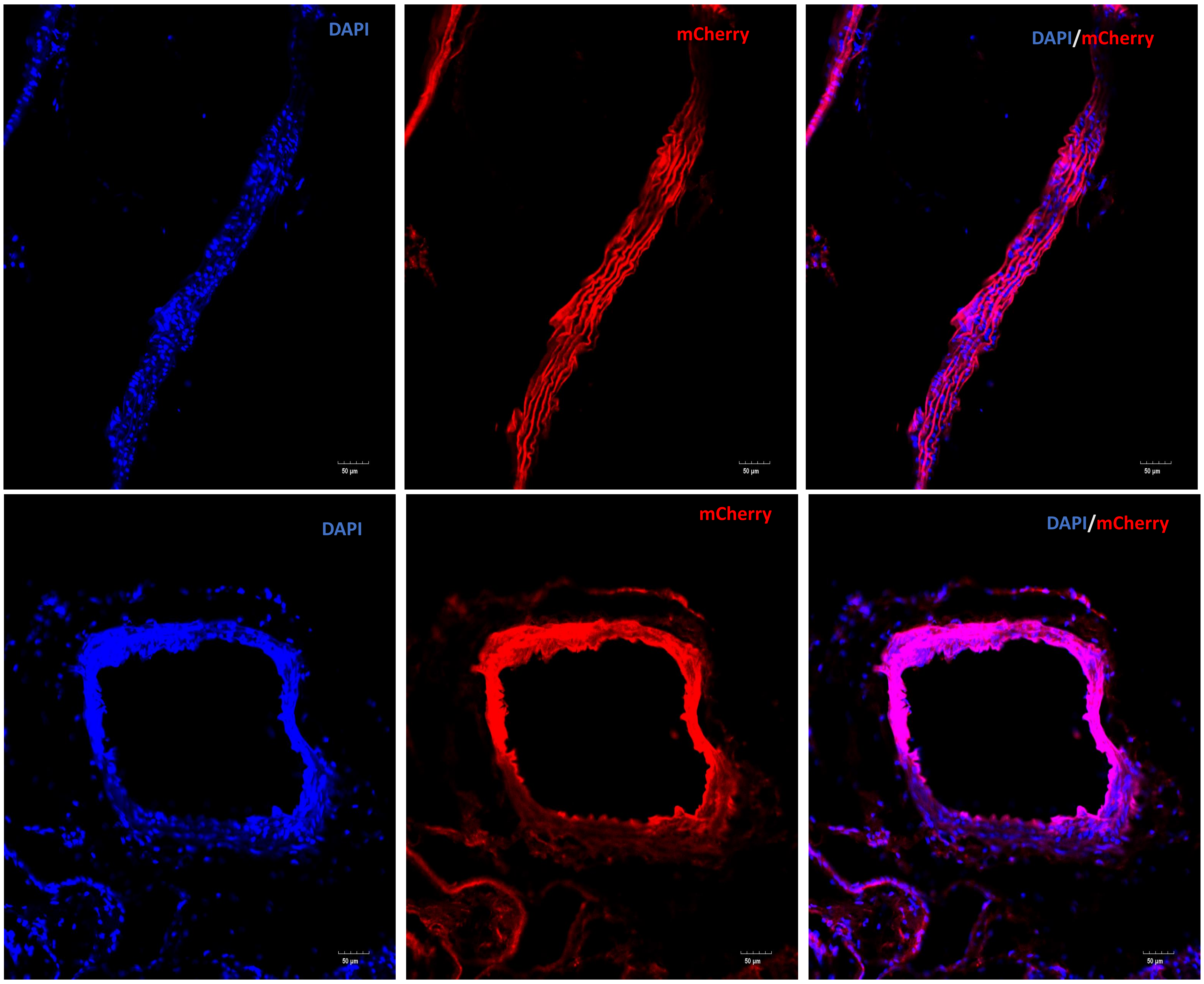

Supplementary Figure 5. VSMC targeting of the AAV9-SM22α-NC or AAV9- SM22α-LCN2. Representative fluorescence images of mCherry in aortic tissue sections from AAV9- SM22α-NC or AAV9-SM22α-LCN2-injected mice. Scale bar:50µm. AAV9, adeno-associated virus 9; VSMC, vascular smooth muscle cell; NC， negative control; SM22α, smooth muscle 22α.

Supplementary Figure 6

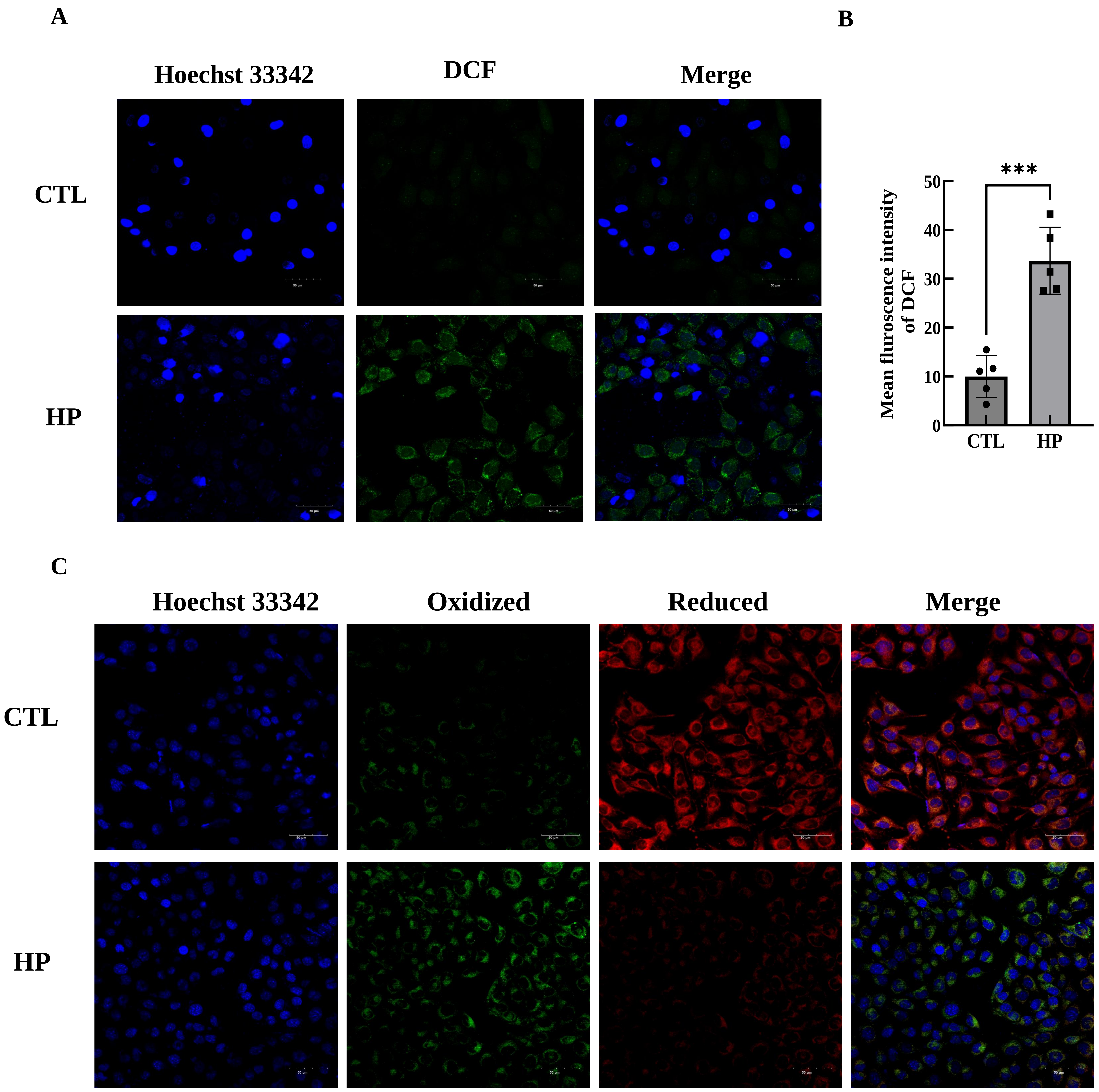

Supplementary Figure 6. Intracellular total ROS and lipid ROS in HP-stimulated VSMCs. A,B. Intracellular total ROS in HP-stimulated VSMCs detected by the fluorescent probe of DCFDA. .Scale bar:50μm. (n=5 per group, $^{***}p < 0.001$ vs. CTL). **C.** Lipid ROS in HP-stimulated VSMCs detected by the fluorescent probe C11-BODIPY$^{581/591}$.Scale bar:50μm. Data are presented as mean ± SD. ROS, reactive oxygen species; HP, high phosphate; VSMCs, vascular smooth muscle cells.

Supplementary Figure 7

A

Gene	sense (5'-3')	Anti-sensse(5'-3')
Negative control	UUCUUCGAACGUGUCACGUTT	ACGUGACACGUUCGGAGAATT
Lcn2-Mus-238	GCACCAUCUAUGAGCUACATT	UGUAGCUCAUAGAUGGUGCTT
Lcn2-Mus-603	GACCAAUGCAUUGACAACUTT	AGUUGUCAAUGCAUUGGUCTT
Lcn2-Mus-356	CCAGUUCACUCUGGGAAAUTT	AUUUCCCAGAGUGAACUGGTT

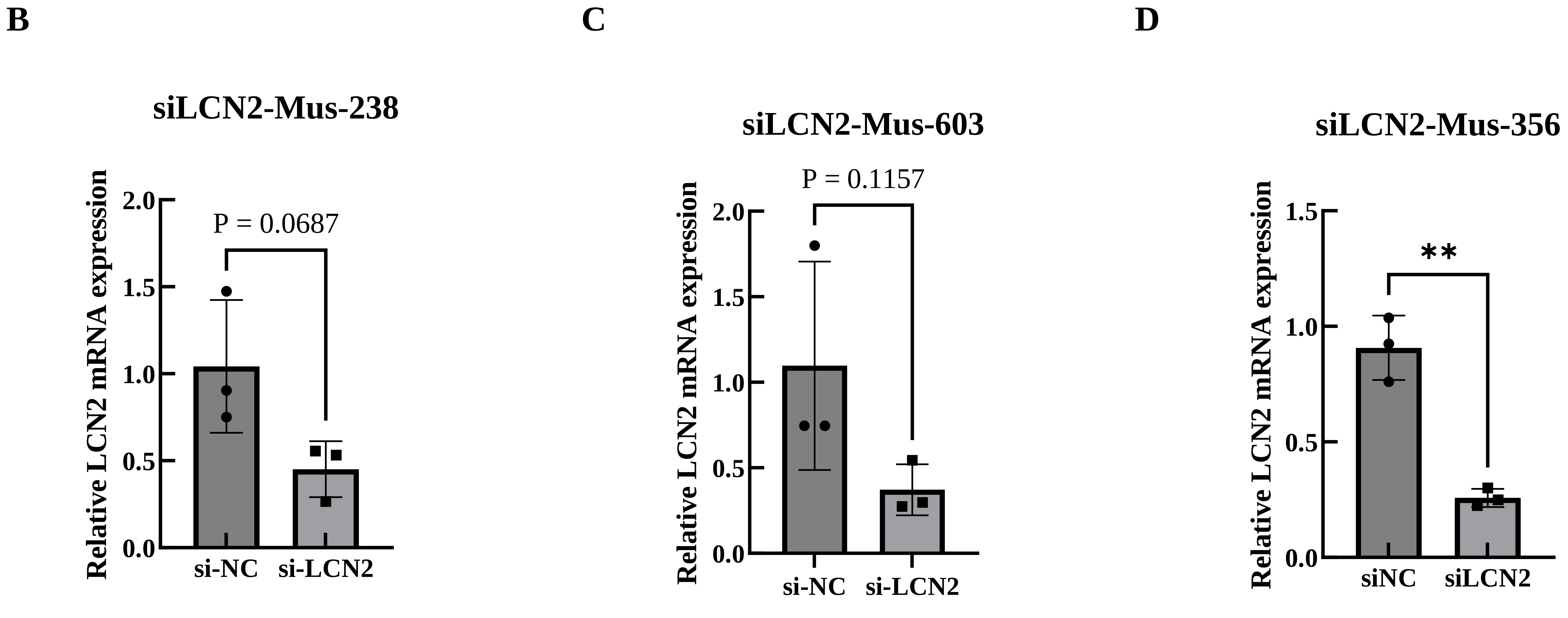

Supplementary Figure 7. The sequences and efficiency results of three siRNAs targeting LCN2. A. The sequences of the negative control siRNA and three siRNAs targeting LCN2. **B.** The knockdown efficiency of the siRNA (LCN2-Mus-238) was determined via qRT–PCR (n=3 per group, *p*=0.0687 vs. siNC). **C.** The knockdown efficiency of the siRNA (LCN2-Mus-603) was determined via qRT–PCR (n=3 per group, *p*=0.1157 vs. siNC). **D.** The knockdown efficiency of the siRNA (LCN2-Mus356) was detected via qRT–PCR. (n=3 per group, $^{**}p<0.01$ vs. siNC).Data are presented as mean ± SD. siRNA, small interfering RNA; qRT–PCR, quantitative real-time polymerase chain reaction; NC, negative control; SD, standard deviation.
