# Supplementary material for: Lipocalin-2 promotes CKD vascular calcification by aggravating VSMCs ferroptosis through NCOA4/FTH1-mediated ferritinophagy
Source: Cell Death Dis. 2024 Nov 29;15(11):865. doi: 10.1038/s41419-024-07260-x (PMC11607329; doi:10.1038/s41419-024-07260-x)

Original Images of Representative Western blot images

Figure 2H

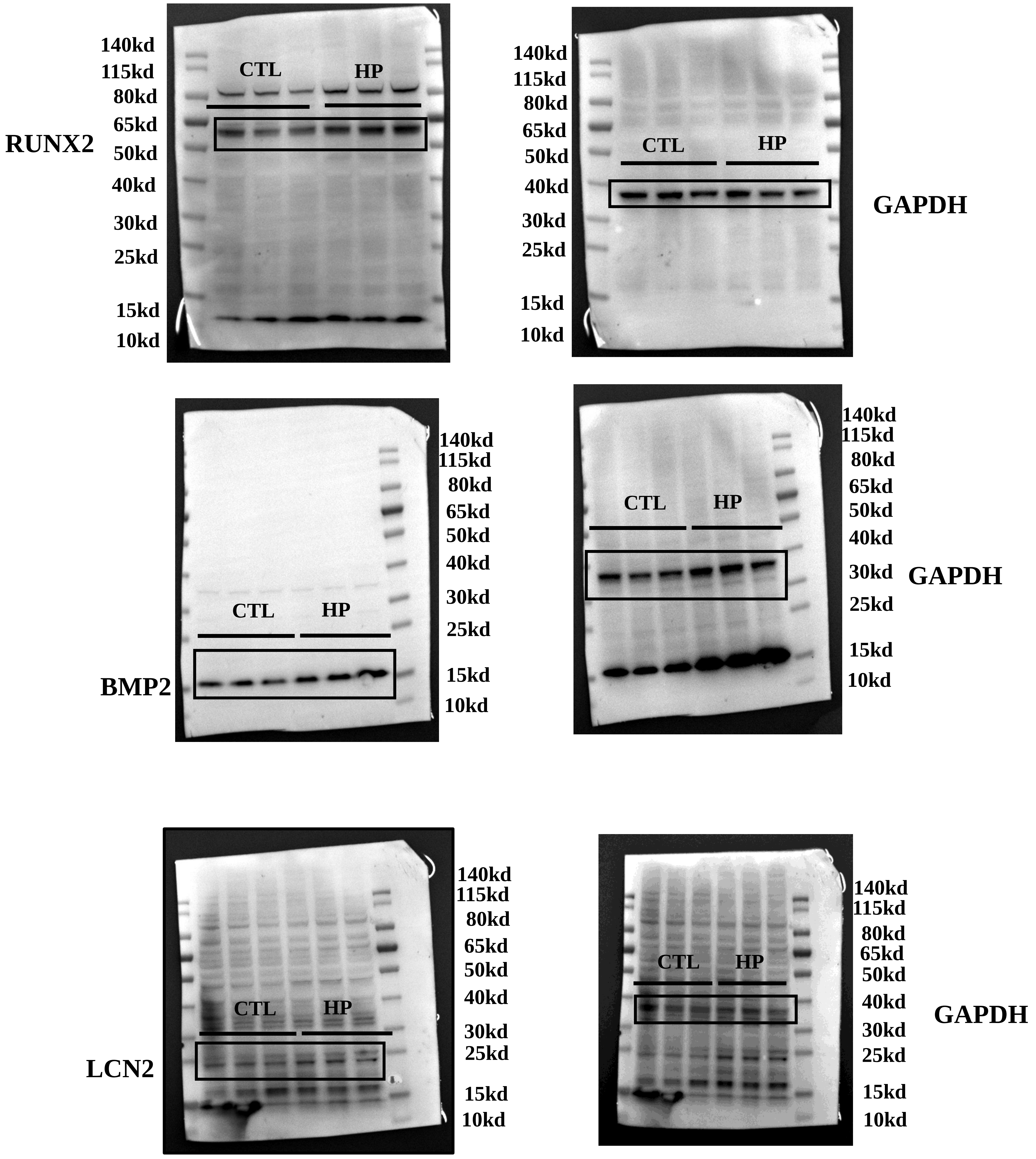

Figure 3H

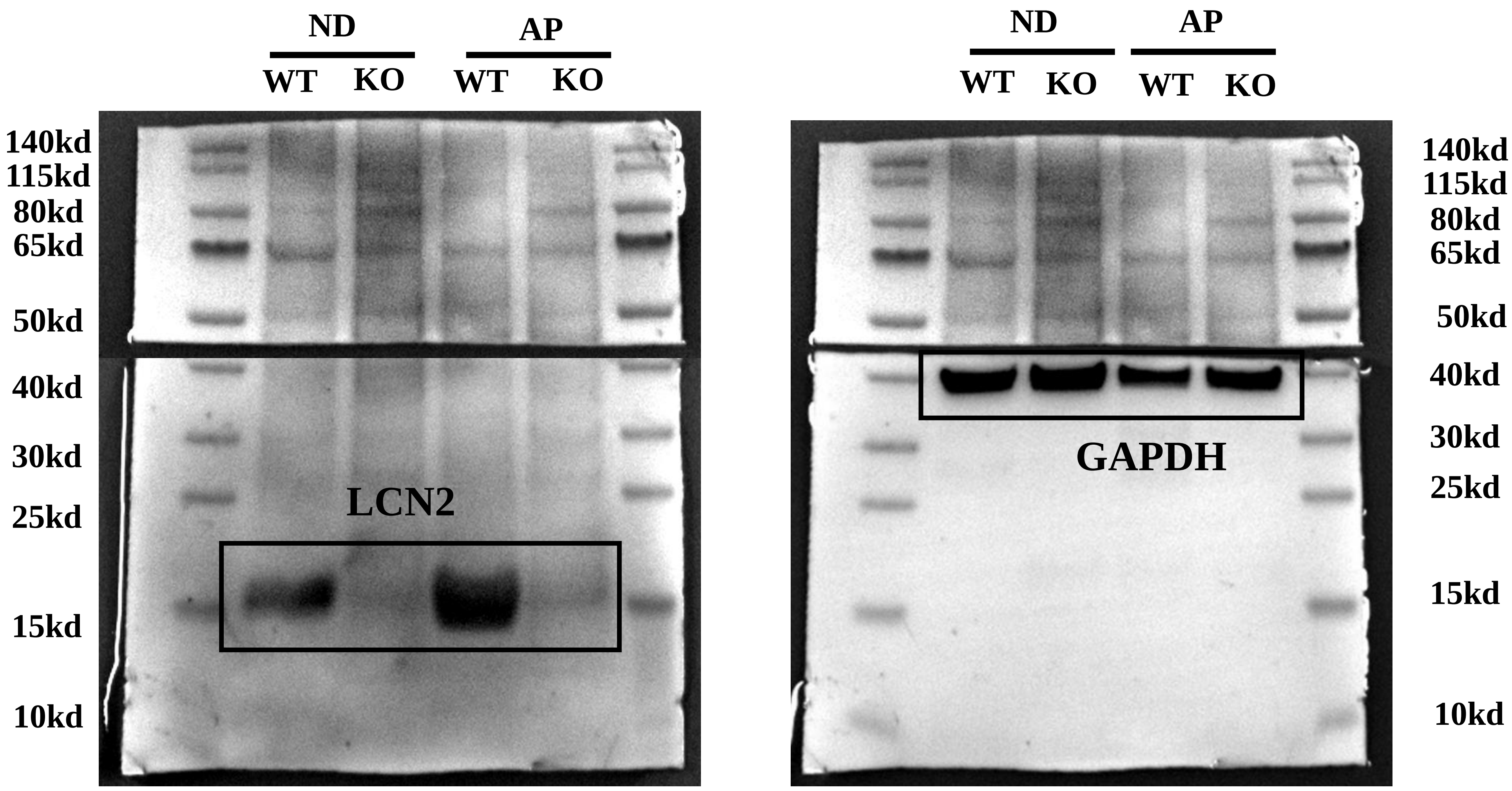
ND
AP
WT
KO
WT
KO
140kd
115kd
80kd
65kd
50kd
40kd
30kd
25kd
15kd
10kd
LCN2
ND
AP
WT
KO
WT
KO
140kd
115kd
80kd
65kd
50kd
40kd
30kd
25kd
15kd
10kd
GAPDH

Figure 4A

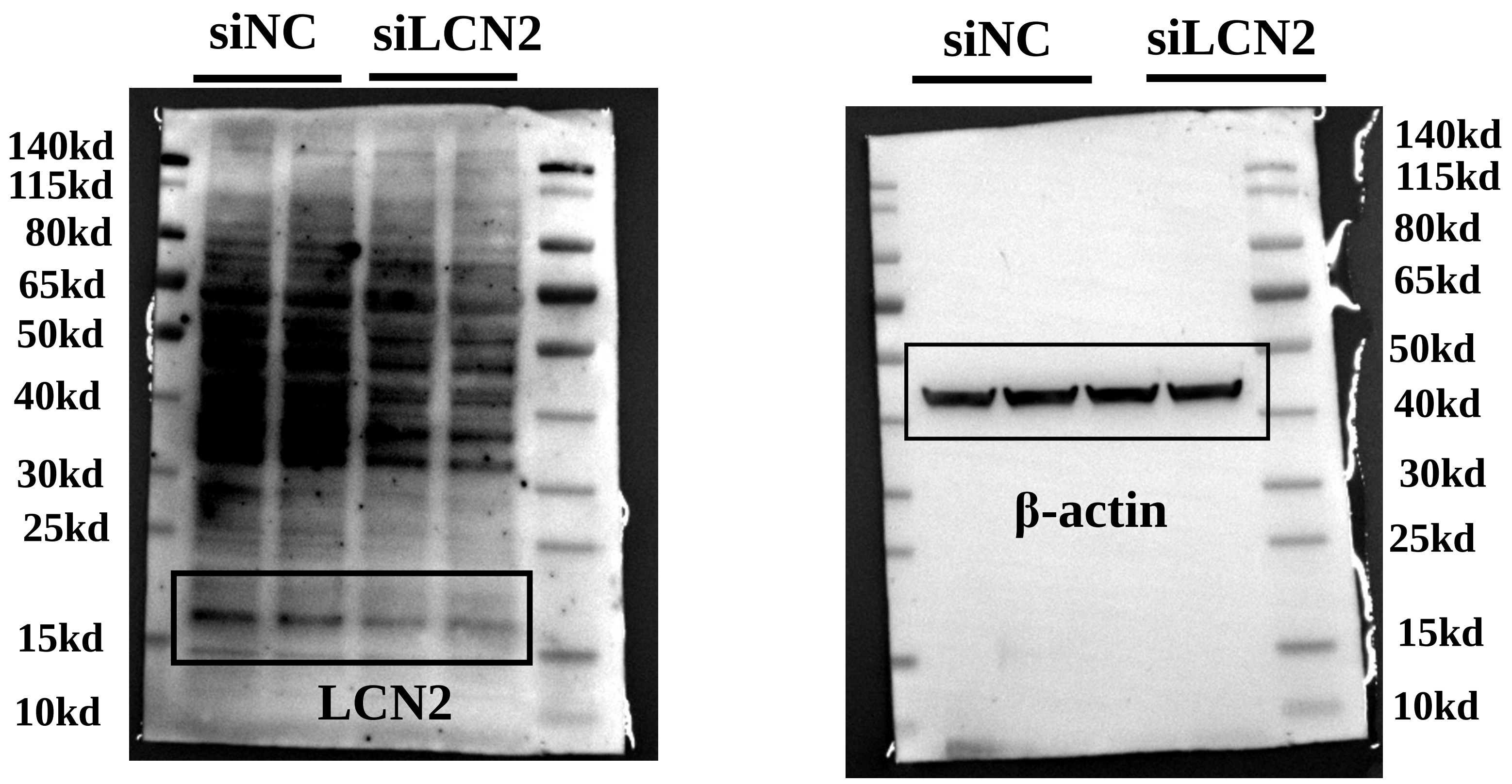

Figure 4G

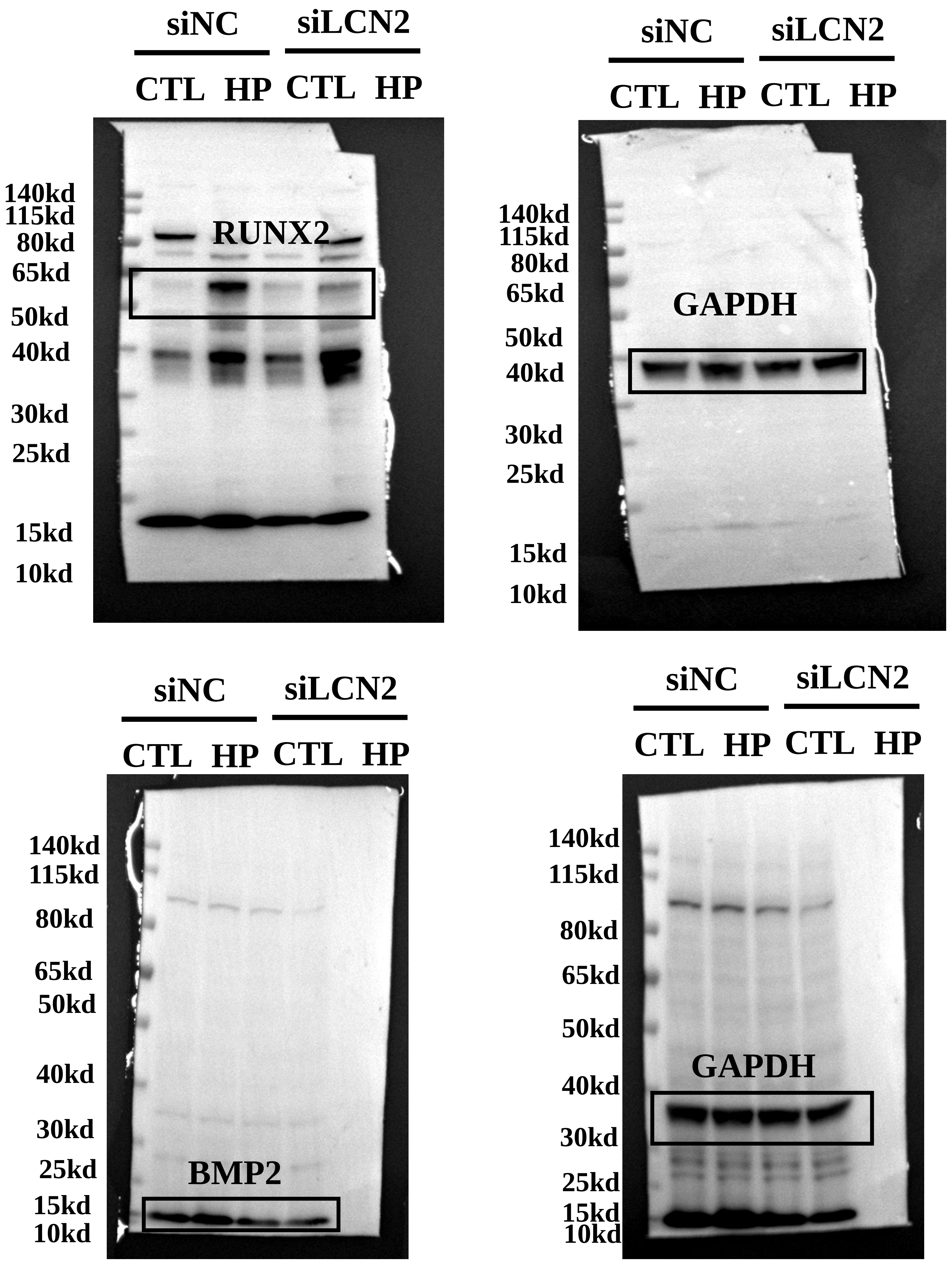

Figure 5F

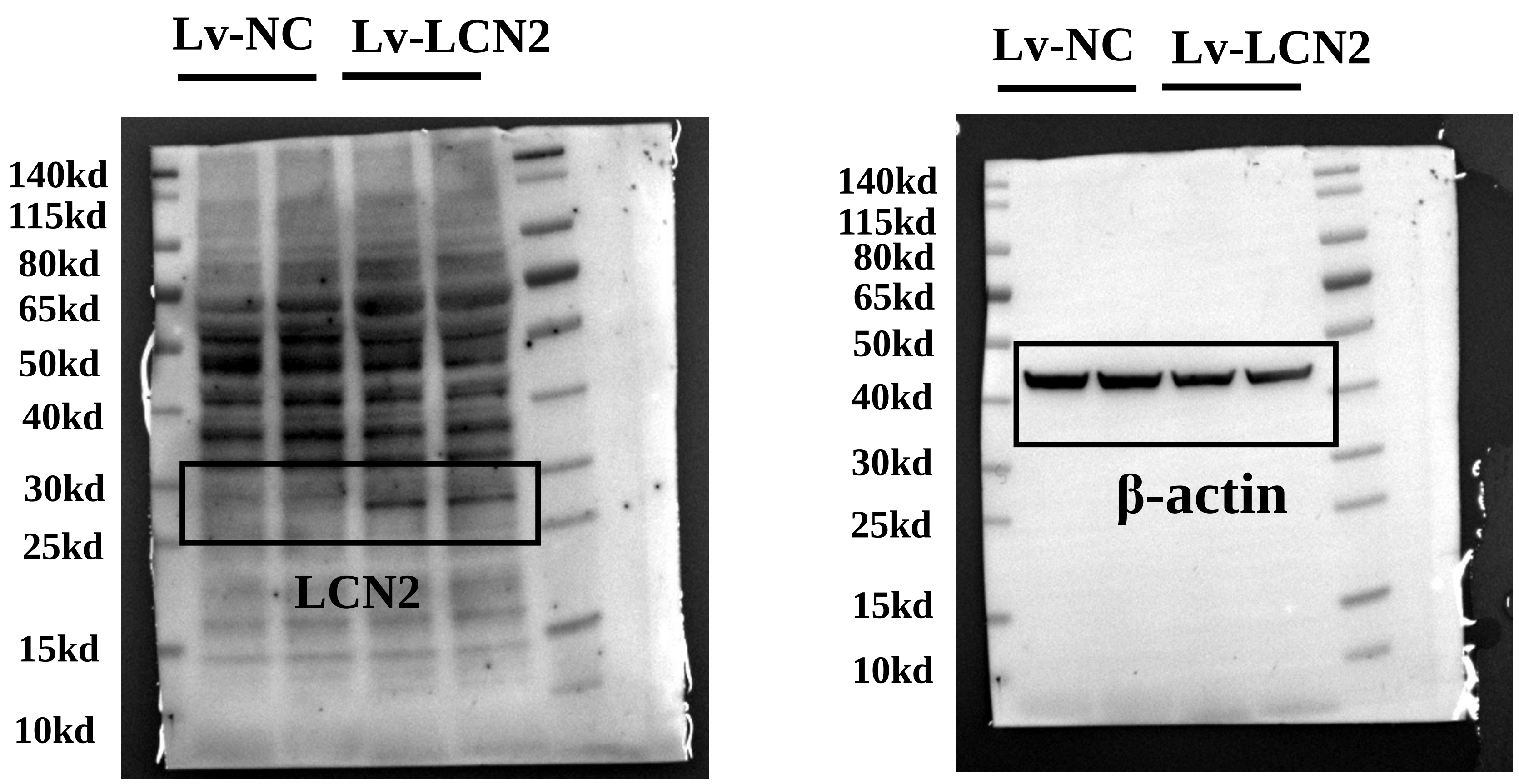
Lv-NC
Lv-LCN2
140kd
115kd
80kd
65kd
50kd
40kd
30kd
25kd
15kd
10kd
LCN2
Lv-NC
Lv-LCN2
140kd
115kd
80kd
65kd
50kd
40kd
30kd
25kd
15kd
10kd
β-actin

Figure 6C

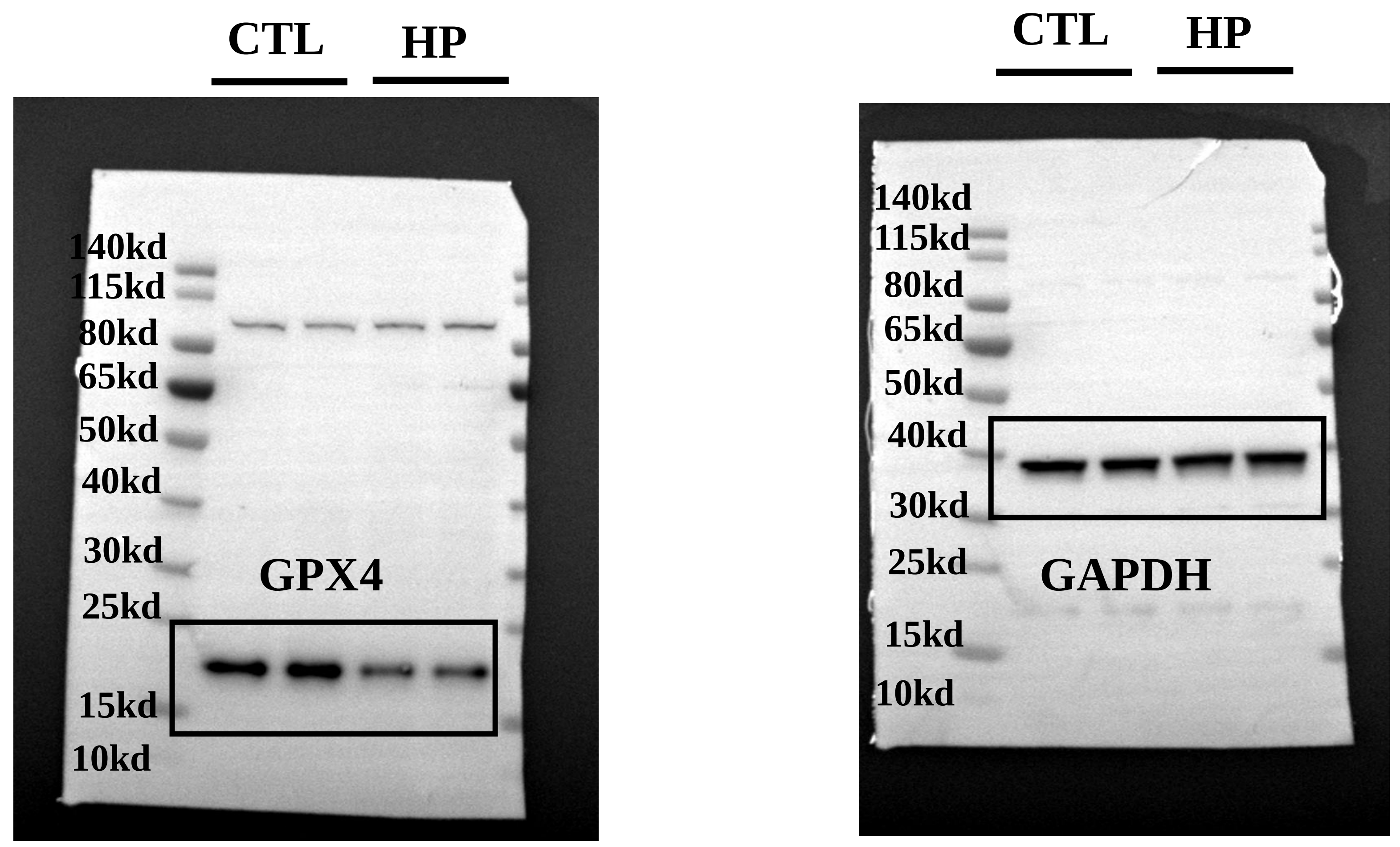

Figure 6L

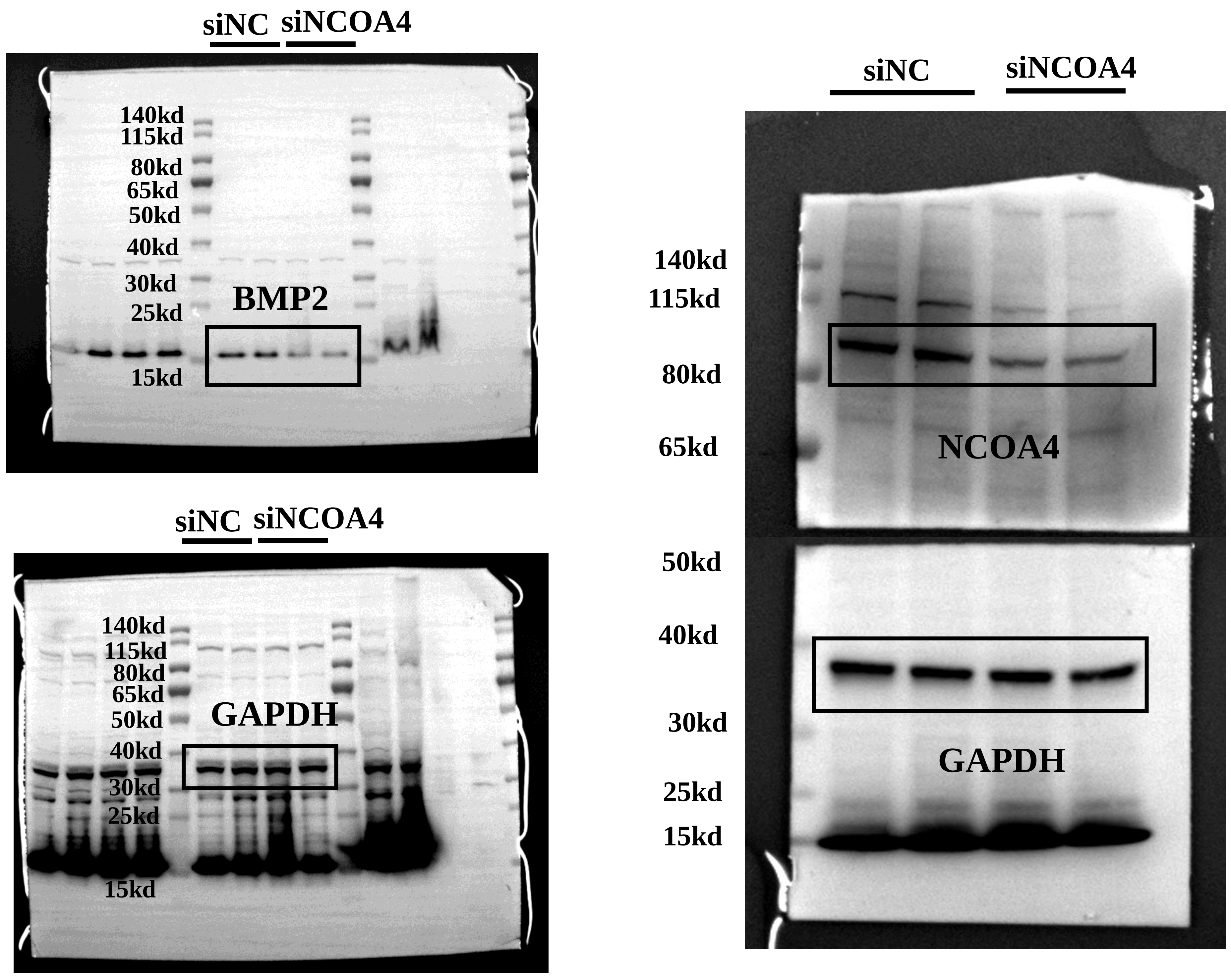

Figure 7J

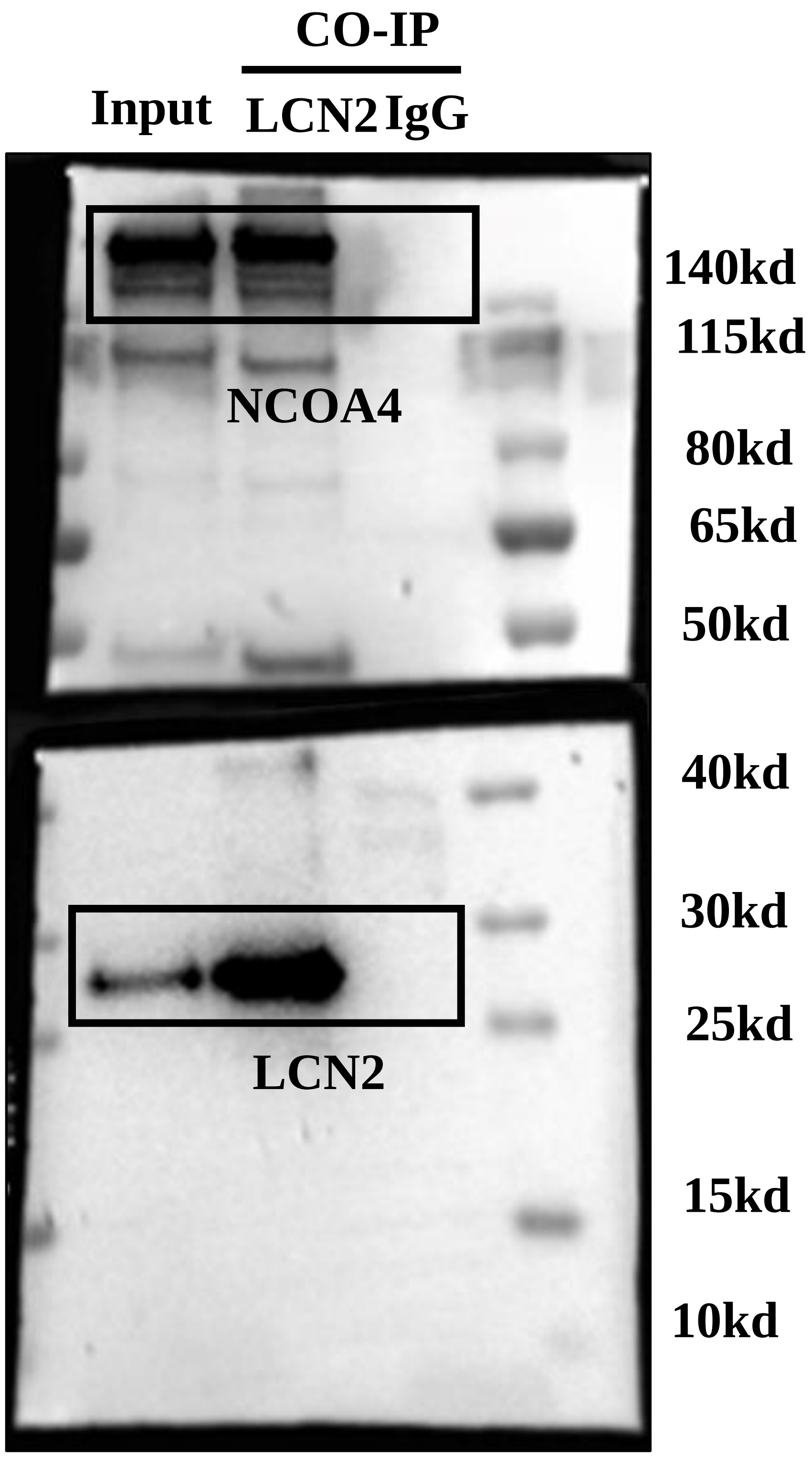

Figure 7K

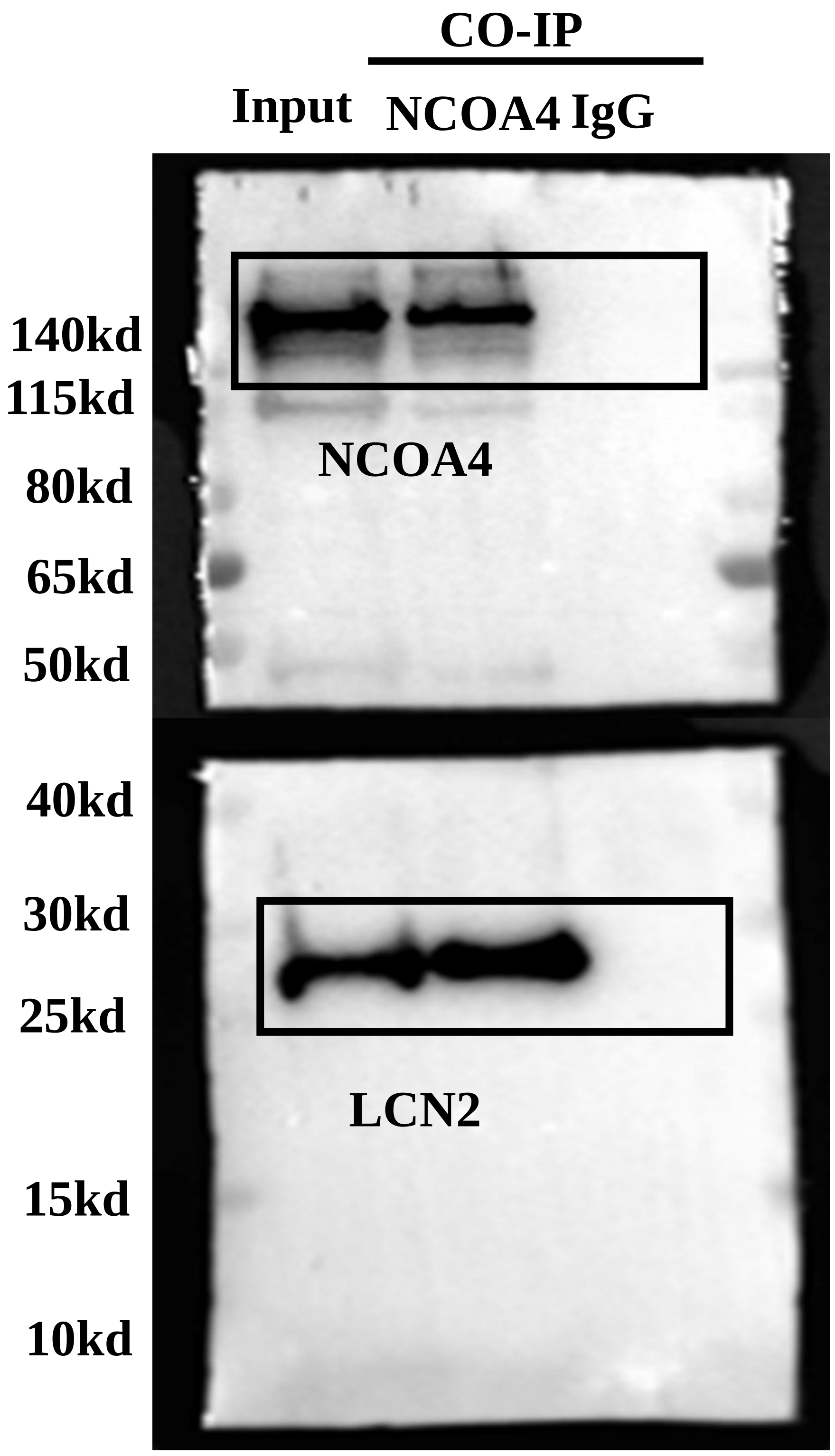

Figure 7L

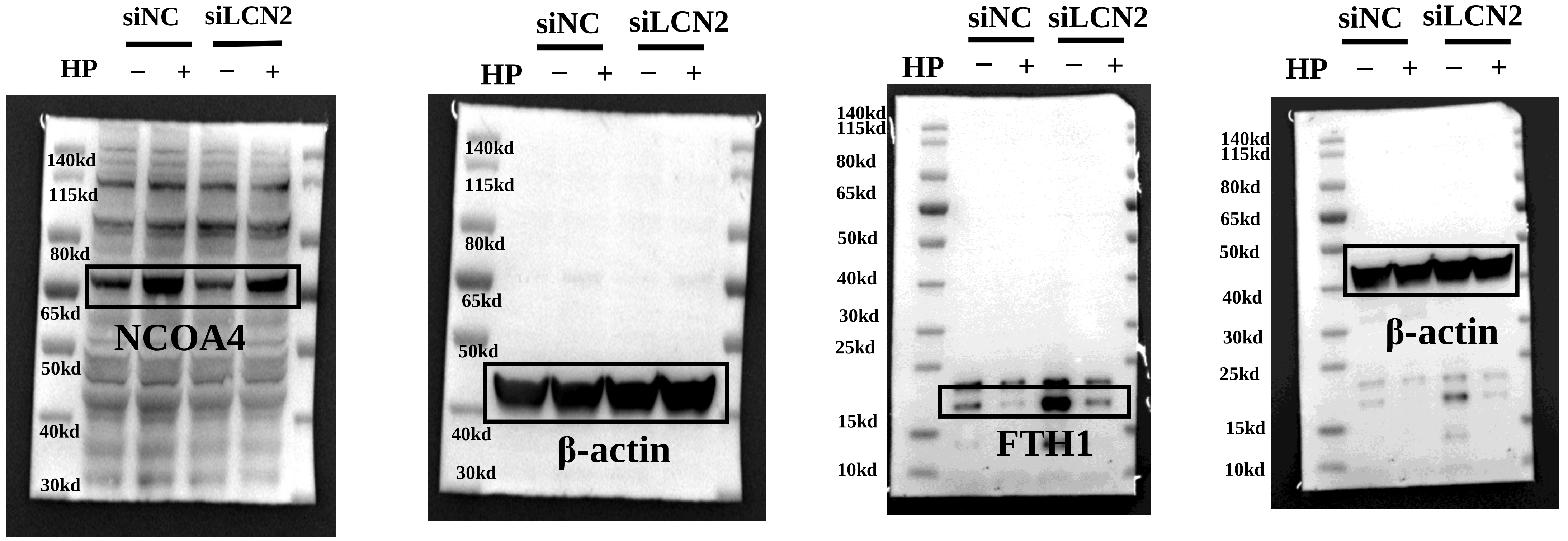

Figure 7O

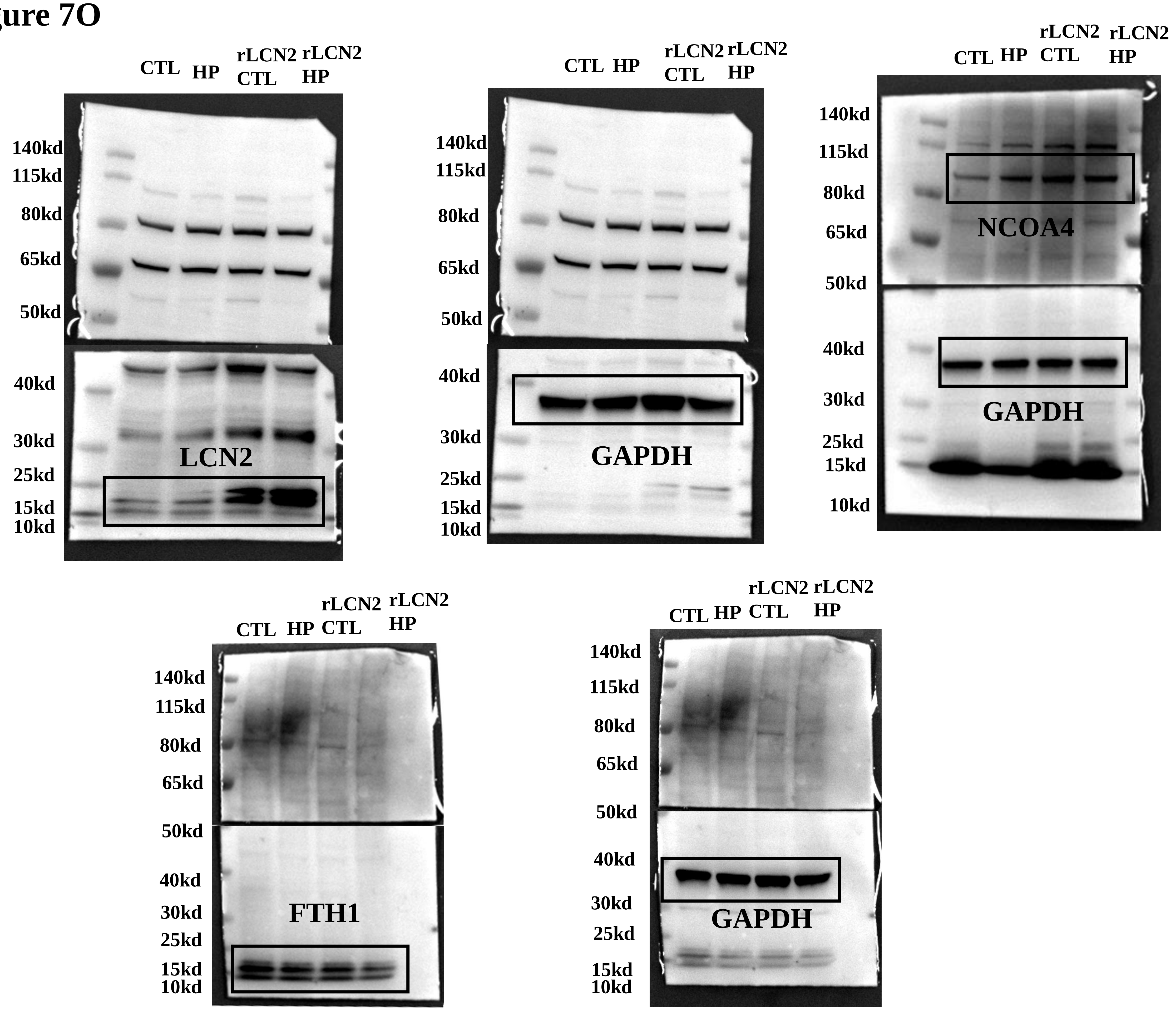

Supplemental Figure 2A

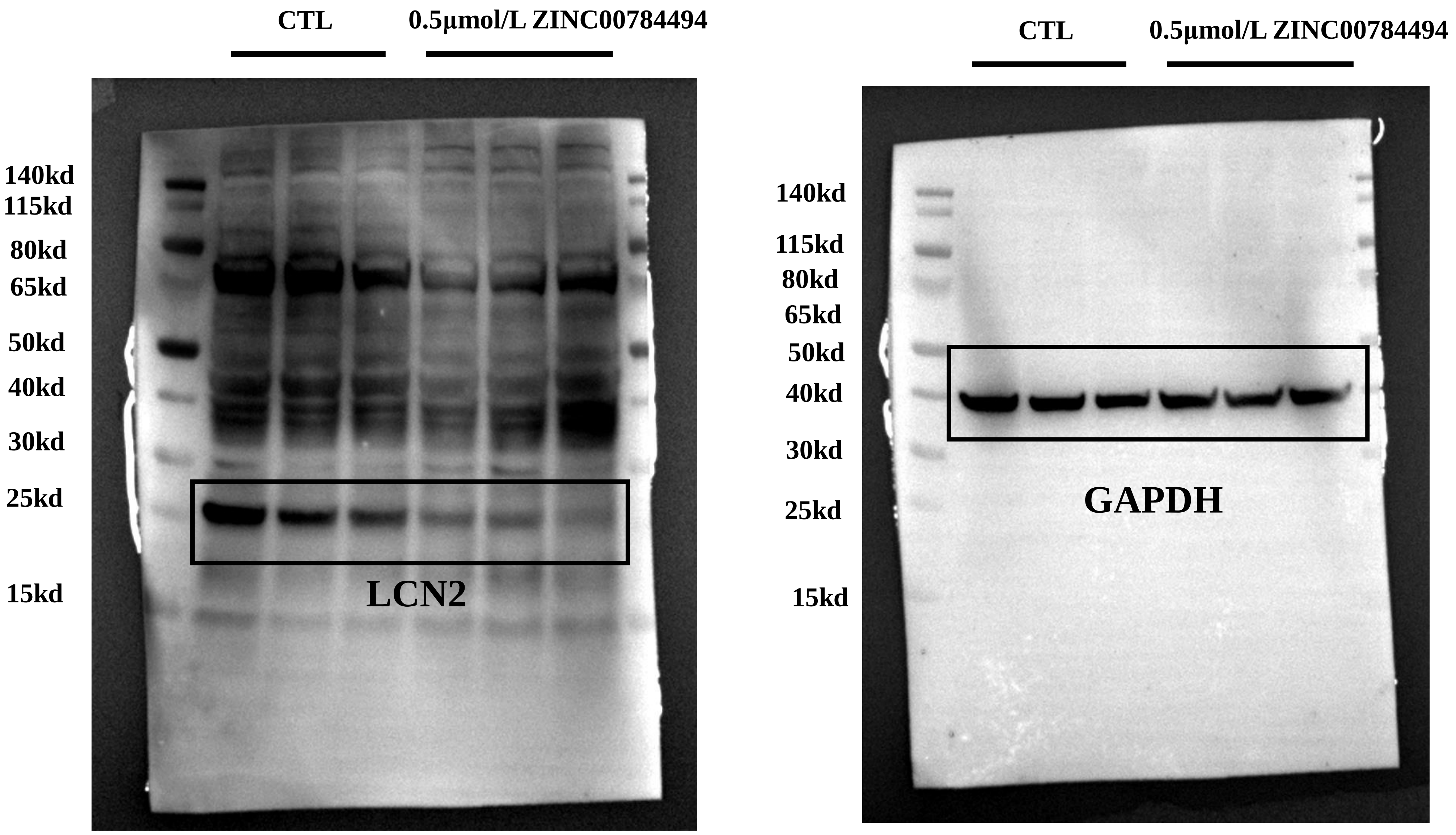

Supplement: Supplementary file 2 — Original images of representative western blot images [file 41419_2024_7260_MOESM2_ESM.pdf]
